# An atlas of larval organogenesis in the European shore crab *Carcinus maenas* L. (Decapoda, Brachyura, Portunidae)

**DOI:** 10.1186/s12983-018-0271-z

**Published:** 2018-07-06

**Authors:** Franziska Spitzner, Rebecca Meth, Christina Krüger, Emanuel Nischik, Stefan Eiler, Andy Sombke, Gabriela Torres, Steffen Harzsch

**Affiliations:** 1 Zoological Institute and Museum, Department of Cytology and Evolutionary Biology, Universität Greifswald, D-17498 Greifswald, Germany; 2 Alfred Wegener Institute, Helmholtz Centre for Polar and Marine Research, Biologische Anstalt Helgoland, D-27498 Helgoland, Germany; 3 Department of Ecology, Environment and Plant Sciences, Stockholm University, Svante Arrhenius väg 20A/F, 11418 Stockholm, Sweden

**Keywords:** Micro-CT, 3D reconstruction, Osmoregulation, Excretion, Sensory systems, Central nervous system, Metamorphosis, Locomotion

## Abstract

**Background:**

The life history stages of brachyuran crustaceans include pelagic larvae of the Zoea type which grow by a series of moults from one instar to the next. Zoeae actively feed and possess a wide range of organ systems necessary for autonomously developing in the plankton. They also display a rich behavioural repertoire that allows for responses to variations in environmental key factors such as light, hydrostatic pressure, tidal currents, and temperature. Brachyuran larvae have served as distinguished models in the field of Ecological Developmental Biology fostering our understanding of diverse ecophysiological aspects such as phenotypic plasticity, carry-over effects on life-history traits, and adaptive mechanisms that enhance tolerance to fluctuations in environmental abiotic factors. In order to link such studies to the level of tissues and organs, this report analyses the internal anatomy of laboratory-reared larvae of the European shore crab *Carcinus maenas*. This species has a native distribution extending across most European waters and has attracted attention because it has invaded five temperate geographic regions outside of its native range and therefore can serve as a model to analyse thermal tolerance of species affected by rising sea temperatures as an effect of climate change.

**Results:**

Here, we used X-ray micro-computed tomography combined with 3D reconstruction to describe organogenesis in brachyuran larvae. We provide a detailed atlas of the larval internal organization to complement existing descriptions of its external morphology. In a multimethodological approach, we also used cuticular autofluorescence and classical histology to analyse the anatomy of selected organ systems.

**Conclusions:**

Much of our fascination for the anatomy of brachyuran larvae stems from the opportunity to observe a complex organism on a single microscopic slide and the realization that the entire decapod crustacean bauplan unfolds from organ anlagen compressed into a miniature organism in the sub-millimetre range. The combination of imaging techniques used in the present study provides novel insights into the bewildering diversity of organ systems that brachyuran larvae possess. Our analysis may serve as a basis for future studies bridging the fields of evolutionary developmental biology and ecological developmental biology.

## Background

**Fig. 1:**
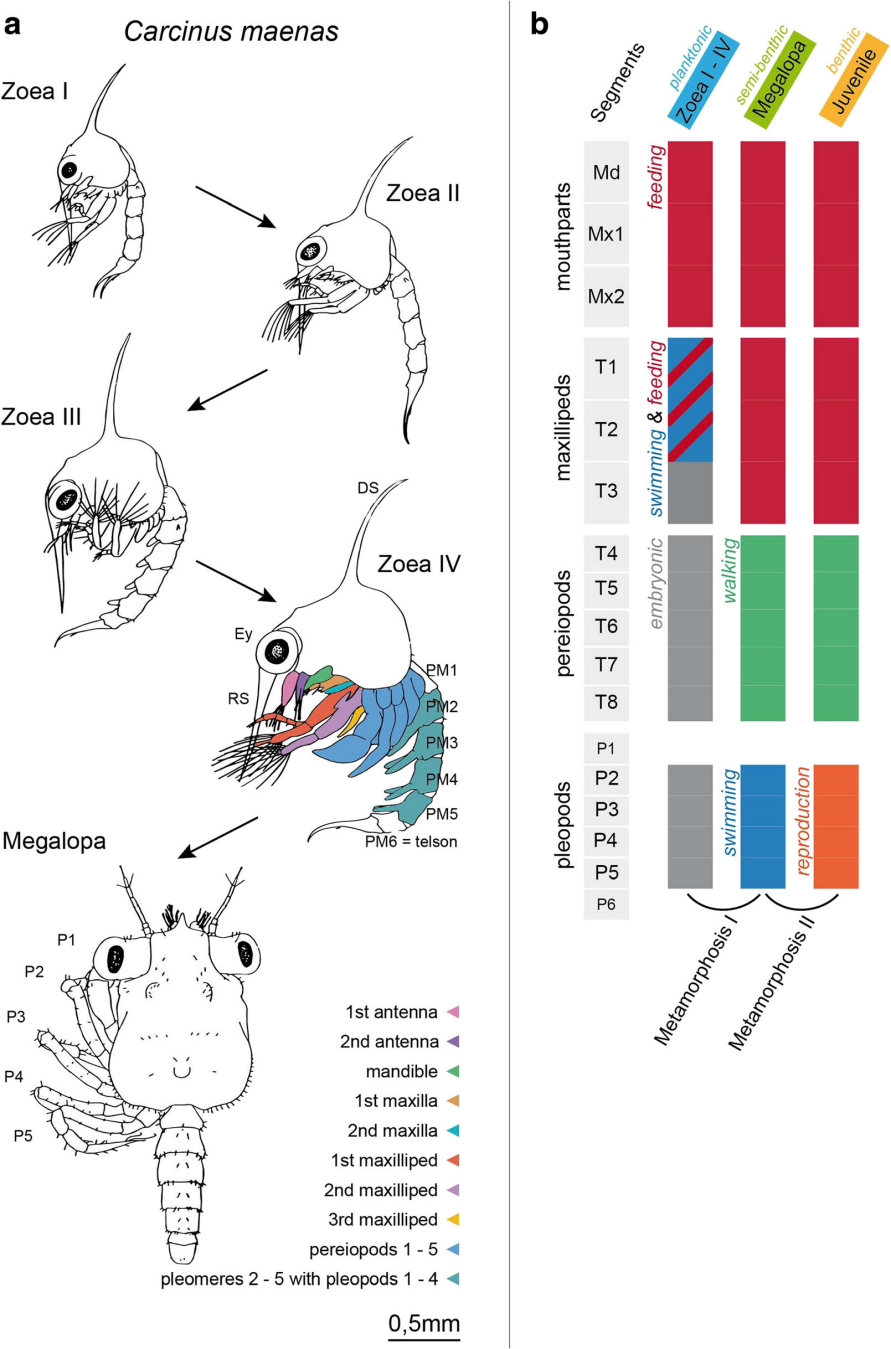
**a** – Larval development of *Carcinus maenas* L. (Decapoda, Brachyura, Portunidae) modified from Rice and Ingle (1975). **b** – ontogenetic change of appendage function during the double metamorphosis (see text for further details). Abbreviations: Ey - compound eye, DS - dorsal spine, Md – mandibular segment, Mx1, Mx2 – segments of 1^st^ and 2^nd^ maxilla, P1–5 - pereiopod one to five, PM1–6 - pleomeres one to six, RS - rostral spine, T1-8 – thoracomeres one to eight

Brachyuran crustaceans display a complex life cycle that includes a pelagic larval phase and a benthic juvenile-adult phase (reviews [1–7]). The adult females carry eggs, from which in most species larvae hatch that are called zoeae and that subsequently develop in the plankton. Larval development includes a series of moults from one zoea instar to the next (Fig. 1a), and may last for several days up to months. The number of zoea instars varies between species and may change within a species depending on environmental factors. For example, decreased salinity causes a delay in larval development of *Neohelice granulata*, including an additional zoeal instar [8]. Zoeae actively feed and display a wide array of adaptations for survival in the pelagic environment concerning their morphology, physiology, behaviour and ecology (reviews [1, 3]). Life in the plankton provides opportunities but also bears risks. Co-occurring plankton organisms provide a rich source of food; however, local and temporal limitations of food availability as well as unsuitable temperatures and salinities represent factors that increase larval mortality (reviews [4, 7]). Furthermore, long periods of growth in the plankton are essential for dispersal and range expansion of brachyuran species and to connect established populations, even if a longer pelagic phase enhances the risk of predation.

Zoeae are known to possess a wide range of organ systems necessary for autonomously surviving and developing in the plankton (Tab. 1) including a sophisticated digestive system, osmoregulatory organs, a well-developed neuromuscular system and a range of sensory organs to detect environmental cues (e.g. light, gravity, temperature, chemical stimuli). They also display a rich behavioural repertoire that allows for responses to variations in environmental key factors: light, hydrostatic pressure, tidal currents, temperature, salinity, and food concentration (reviews [7, 9, 10]). For example, zoeae can control their position within the water column and, by distinct vertical migration behaviour, use tidal currents for offshore transport (reviews [7, 11, 12]). In addition, they use perceived chemical cues from their conspecifics to identify suitable habitats to metamorphose and recruit (reviews [4, 7, 9, 10, 13]). For many decades, brachyuran larvae have served as distinguished models in the field of Ecological Developmental Biology (EcoDevo; reviews [4, 7]). Laboratory and field studies on the development of brachyuran larvae have fostered our understanding of diverse ecophysiological aspects such as phenotypic plasticity in developmental traits, heterochrony in developmental patterns, carry-over effects on life-history traits, and adaptive mechanisms that enhance tolerance to fluctuations in environmental abiotic and biotic factors. Furthermore, a diverse range of biological topics has been analysed using brachyuran larvae as models including aspects of the physiology of aquatic-terrestrial and marine-limnic transitions, dispersal potential of invasive species, adaptive significance of abbreviated development, and effects of acclimation (reviews [4, 7]) but also the effects of environmental change-induced abiotic stress on ontogenetic stages of marine organisms [14].

**Table 1:** Studies on larval organogenesis in representatives of Pleocyemata

General internal anatomy	
*Cancer anthonyi*	Trask 1974 [21]
*Portunus trituberculatus*	Nakamura 1990 [22]
Mouthparts and digestive tract	
*Maja brachydactyla*	Castejon et al. 2015 [89]
*Hyas araneus*	Storch and Anger 1983 [72], Höcker 1988 [82]
*Scylla olivacea*	Jantrarotai et al. 2005 [137]
*Scylla serrata*	Li and Li 1995 [138], 1998 [139], Lumasag et al. 2007 [140]
*Ucides cordatus*	Abrunhosa et al. 2003 [91]
*Dyspanopeus sayi*	Castejon et al. 2015 [92]
*Sesarma curacaoense*	Melo et al. 2006 [93]
several Brachyura	Geiselbrecht and Melzer 2010 [20]
*Ranina ranina*	Minagawa and Takashima 1994 [88]
*Menippe mercenaria*	Factor 1982 [23]
*Paralithodes camtschaticus*	Abrunhosa and Kittaka 1997 [96]
*Homarus americanus*	Anger et al. 1985 [98], Sasaki et al. 1986 [141], Factor 1981 [17], Biesot and McDowell 1995 [97]
*Procambarus fallax* f. *virginalis*	Vogt 1994 [71], 2008 [49, 50]
*Jasus edwardsii*	Nishida et al. 1990 [142]
*Macrobrachium amazonicum*	Batel et al. 2014 [143]
*Palaemon elegans*	Batel et al. 2014 [143]
Osmoregulatory epithelia	
*Carcinus maenas*	Cieluch et al. 2004 [43], Hong 1988 [77]
*Eriocheir sinensis*	Cieluch et al. 2007 [44],
*Callianassa jamaicense*	Felder et al. 1986 [144]
*Astacus leptodactylus*	Lignot et al. 2005 [29]
several Brachyura and Anomala	Hong 1988 [77]
*Homarus americanus*	Lignot and Charmantier 2001 [31]
*Palaemontes argentinus*	Leone et al. 2012 [134], Ituarte et al. 2016 [45]
*Litopenaeus stylirostris*	Pham et al. 2016 [145]
Antennal glands	
*Astacus leptodactylus*	Khodabandeh et al. 2005 [80, 81]
*Homarus gammarus*	Khodabandeh et al. 2006 [108]
*Macrobrachium amazonicum*	Boudour-Boucheker et al. 2013 [79]
*Palaemontes argentinus*	Ituarte et al. 2016 [45]
Integument and tegumental glands	
*Hyas araneus*	Höcker 1988 [82]
*Sesarma haematocheir*	Ikeda et al. 2004 [32]
multiple species	Freemann 1993 [146]
Y organ	
*Cancer anthonyi*	McConaugha 1980 [132]
*Hyas araneus*	Höcker 1988 [82]
Eyestalk neuroendocrine centres	
*Cancer anthonyi*	McConaugha 1980 [132]
*Homarus gammarus*	Rotllant et al. 1994 [26], 1995 [27]
Compound eyes	
*Callinectes sapidus*	Cronin et al. 1995 [147]
*Carcinus maenas*	Harzsch and Dawirs 1996 [25]
*Hemigrapsus sanguineus*	Charpentier and Cohen 2015 [148]
*Rhithropanopeus harrisii*	Charpentier and Cohen 2015 [148]
*various Anomala*	Fincham 1988 [149]
*Panulirus longipes*	Meyer-Rochow 1975 [150]
*Procambarus clarkii*	Hafner and Tokarski 1998 [151]
Aesthetascs	
*Carcinus maenas*	Ekerholm and Hallberg 2002 [83]
*Cherax destructor*	Sandeman and Sandeman 1990 [152]
Structure of the CNS and neurogenesis	
*Carcinus maenas*	Harzsch and Dawirs 1993 [24]
*Hyas araneus*	Harzsch and Dawirs 1994 [36], 1995 [41], Harzsch et al. 1998 [38]
*Pachygrapsus marmoratus*	Geiselbrecht and Melzer 2013 [30]
*Porcellana platycheles*	Geiselbrecht and Melzer 2013 [30]
*Cherax destructor*	Sullivan and MacMillan 2001 [40]
*Homarus americanus*	Helluy et al. 1995 [28], Harzsch et al. 1998 [38]
*Hippolyte inermis*	Geiselbrecht and Melzer 2013 [30]
Immunolocalization of neuroactive substances in the CNS	
*Carcinus maenas*	
moult inhibiting hormone, crustacean cardioactive peptide, crustacean hyperglycemic hormone	Webster and Dircksen 1991 [130], Chung and Webster 2004 [131]
*Hyas araneus*	
serotonin	Harzsch and Dawirs 1995 [41]
RFamide	Harzsch and Dawirs 1996 [42]
*Astacus leptodactylus*	
crustacean hyperglycemic hormone	Gorgels-Kallen and Meij 1985 [153]
*Cherax destructor*	
serotonin	Sandeman and Sandeman 1990 [152], Helluy et al. 1993 [154]
GABA, glutamate	Foa and Cooke 1998 [155]
*Homarus americanus*	
serotonin	Beltz et al. 1990 [156], 1992 [157], Helluy et al. 1993 [154], Harzsch 2003b [158]
proctolin	Beltz et al. 1990 [156], 1992 [157]
dopamine	Cournil et al. 1995 [159]
octopamine	Schneider et al. 1996 [160]
nitric oxide/cyclic guanosine mono-phosphate	Scholz et al. 1998 [161], Benton et al. 2007 [162]
pigment dispersing hormone	Harzsch et al. 2009 [163]
various neuropeptides	Pulver and Marder 2002 [164]
*Homarus gammarus*	
gonad-inhibiting hormone	Rotllant et al. 1993 [165], 1995 [27]
*Procambarus fallax* f. *virginalis*	
serotonin	Zieger et al. 2013 [166]
histamine	Rieger and Harzsch 2008 [167]

The last zoeal instar of Brachyura metamorphoses into a semi-benthic larva, the Megalopa, the last larval stage in brachyurans (Fig. 1a). This moult is frequently designated as the first metamorphosis. The Megalopa gradually settles on the sea bottom where in a second metamorphosis it moults to an adult-like benthic juvenile. Both metamorphoses are associated with distinct changes in habitat, behaviour, locomotion, feeding, morphology, and ecology (reviews [1, 3–6, 15]). This complex life history involves developmental transformations of the cephalic, thoracic and pleonal appendages as summarized in Fig. 1b. In planktonic zoeae, in addition to handling food items, the first and second maxillipeds also serve a natatory function that is fulfilled by their exopods. During the first metamorphosis, the maxillipeds lose the exopods and with it, their locomotor function and will exclusively serve as part of the feeding apparatus. The pereiopods and pleopods gradually emerge as non-functional embryonic anlagen in the zoeae to become functional for locomotion during the first metamorphosis, corresponding to the requirements of a transition from the pelagic to the benthic life style that takes place during the megalopa stage. The Megalopa of Brachyura can still swim using its pleopods that bear long setae and can use tidal-currents for onshore transport. After the second metamorphosis to the juvenile stage, the organsims become completely benthic and their pleopods lose the natatory function (Fig. 1b) to become part of the reproductive system as copulatory organs and as attachment sites for the extruded mass of fertilized eggs.

**Fig. 2:**
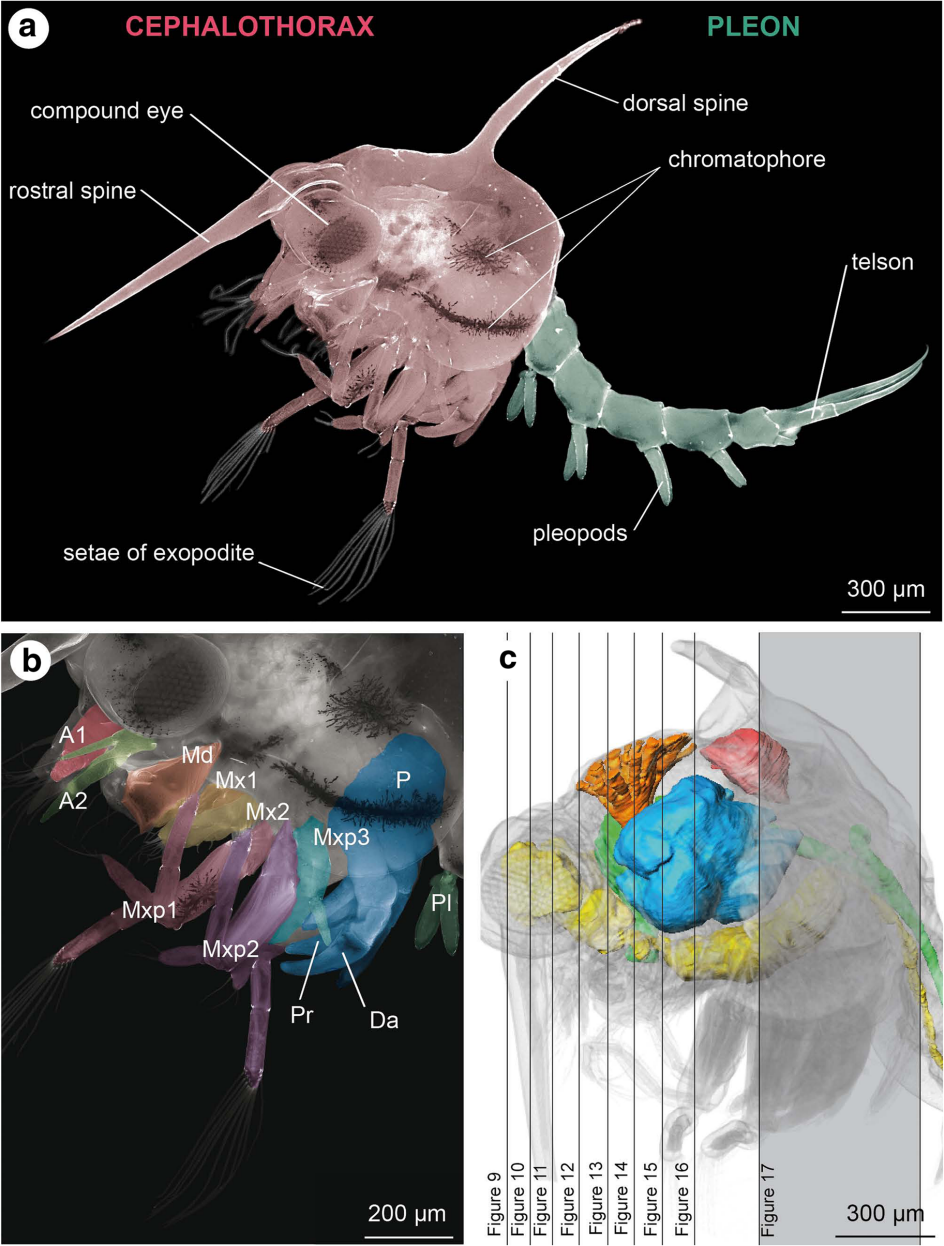
**a -** lateral view of a Zoea IV (autofluorescence of cuticle). **b** - higher magnification of specimen in A showing cephalic and thoracic appendages, the colour code corresponds to Fig. 1A. **c**: Zoea IV, 3D-reconstruction of selected organs (colour code see Fig. 3) to indicate the sectioning planes shown in Figs. 9, 10, 11, 12, 13, 14, 15, 16 and 17. Abbreviations: A1 - first antenna, A2 - second antenna, Da - dactylus of the first pereiopod (chela), Md - mandible, Mx1 - first maxilla, Mx2 - second maxilla, Mxp1 - first maxilliped, Mxp2 - second maxilliped, Mxp3 - third maxilliped, P - pereiopod anlagen, Pl - pleopod anlagen, Pr - Propodus of the first pereiopod (chela)

The external morphology of brachyuran larvae has been documented for an extraordinarily wide range of species for many decades, using light microscopy and by line drawings (Fig. 1; [1, 2, 5, 16]), as well as scanning electron microscopy [17–20]. Decapod crustacean larvae provide the fascinating opportunity to study the wealth of organ systems of the adult bauplan compressed into a tiny but autonomous organism that fully fits under the microscope. Nevertheless, and despite their outstanding role as models in ecological developmental biology, our current knowledge on the internal anatomy of brachyuran larvae is still rather limited, the most important resources being studies on *Cancer anthonyi* [21] and *Portunus trituberculatus* [22]. Other techniques to analyse anatomical aspects of the larvae of decapod crustaceans include for example semi-thin sectioning of resin embedded specimens [23–29], three-dimensional reconstruction of histological data [30], transmission electron microscopy [26, 31–34], DiI labelling combined with confocal laser-scan microscopy [35], *in vivo* incubation with mitosis markers [36–39], nuclear labelling with a DNA markers [40]) and immunohistochemical localization of neuronal antigens [41, 42] and ion pumps within transport epithelia [43–45]. Table 1 summarizes studies on the anatomy of developing organ systems, limited to representatives of the Pleocyemata, but including studies that synthesize data on the transition from embryos to larvae. Furthermore, aspects of early embryogenesis in decapod crustaceans have been summarized in a number of contributions [46–56] and will not be further discussed here.

This study sets out to provide a comprehensive overview of the internal anatomy of brachyuran larvae. We selected laboratory-reared larvae of the European shore crab *Carcinus maenas* L. (Decapoda, Brachyura, Portunidae), a species that has a native distribution extending across most European waters from Norway to Mauritania [57]. This species has attracted attention because it has invaded five temperate geographic regions outside of its native range [58] and can serve as a model to analyse thermal tolerance of species impacted by rising sea temperatures as an effect of climate change [59]. The external morphology of *C. maenas* larvae, including the larval appendages, has already been thoroughly documented [60, 61]. They can be reared under controlled conditions in the laboratory [62, 63], an essential requirement for an animal to serve as a model in developmental biology. Consequently, larval elemental composition, respiration rates and energy balances have been analysed during development under optimal conditions for this species ([64]), and multiple factors that affect larval growth and feeding rates, such as temperature and food availability have been examined [65–68]. Here, for the first time, we use X-ray micro-computed tomography combined with 3D reconstruction to describe organogenesis from the first to the last larval stage in this species. Classical histology was used to analyse the anatomy of selected organ systems. We aimed to provide a detailed atlas of the larval internal organization to complement the existing descriptions of the external morphology. Our analysis may serve as a basis for future studies bridging the fields of Evolutionary Developmental Biology and Ecological Developmental Biology.

## Results

### External Morphology

**Fig. 3:**
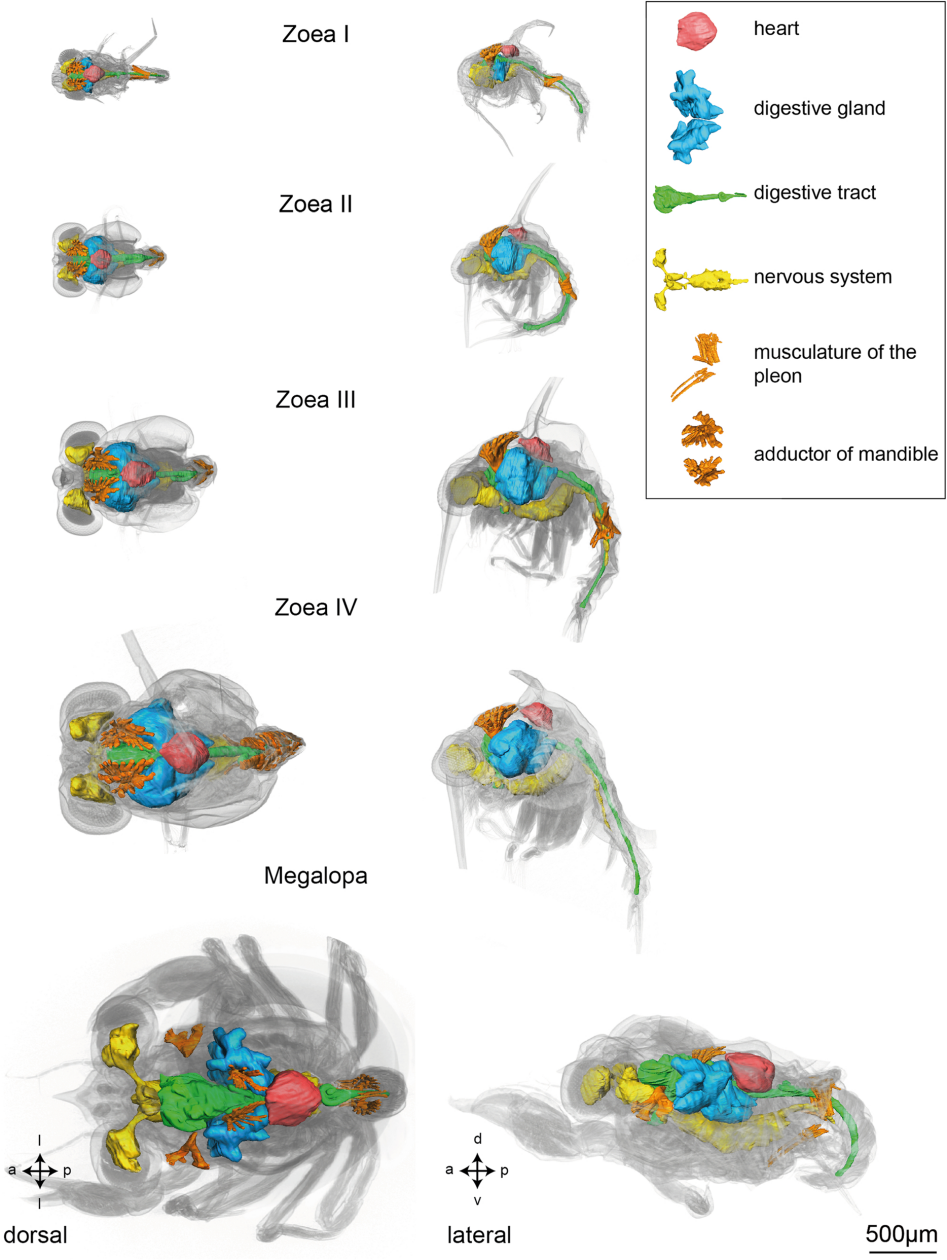
Larval morphology and organogenesis from the Zoea I to the Megalopa based on micro-CT analyses, dorsal views (left panels) and lateral views (right panels). Isosurface views of the cuticle merged with 3D reconstructions of selected organ systems. Crossed arrows indicate the orientation (a - anterior, d - dorsal, l - lateral, p - posterior, v - ventral)

As is typical of all portunid zoeae, the cephalothorax of the *C. maenas* Zoea IV is armed with a characteristic rostral and a dorsal spine (Fig. 2a; and [60, 61]). In the Zoea I, the compound eyes are sessile, but after moulting to the Zoea II they are stalked. In bright field and epifluorescence images, chromatophores embedded within the epidermis can be clearly seen, most prominently at the lateral carapace, the proximal podomeres of maxillipeds, and sometimes also in the pleon (Fig. 2a). The functional cephalic appendages of all zoeal stages comprise the first and second antennae as well as the mouthparts, which include the well-developed and strongly calcified mandibles, followed by the first and second pair of maxillae (Fig. 2b). In the zoeal stages, the first and second maxillipeds display well-developed exopods with long setae at their distal tips, in addition to the endopods (Fig. 2b). The third maxillipeds are not yet functional at hatching but gradually form in the Zoea III and IV where they become visible as conspicuous tissue buds. The anlagen of the thoracic limbs of thoracomere four to eight, the pereiopods, also emerge as small tissue buds in the Zoea II and they continue to grow during the Zoea III. In the Zoea IV, individual podomeres can be distinguished and the first pereiopod (thoracomere four) already displays the propodus and the dactylus of the chela (Fig. 1b). The last segment of the pleon, the telson, in the Zoea IV bears the elongated spine-like uropods and distinct pleopod anlagen are visible on pleomeres number two to five (Figs. 2a, 3). The pleopod anlagen emerge as small buds in the Zoea II and become fully functional in the Megalopa (Fig. 3). Many morphological features of the zoea stages I to IV remain unchanged but the larvae increase in size (Fig. 3). On the contrary, the first metamorphic moult to the Megalopa coincides with major transformations in morphology. The cephalothorax attains the typical adult crab habitus (Fig. 3) and the Megalopa looses the dorsal spine completely, but a small tapered rostrum remains.

### Digestive System

**Fig. 4:**
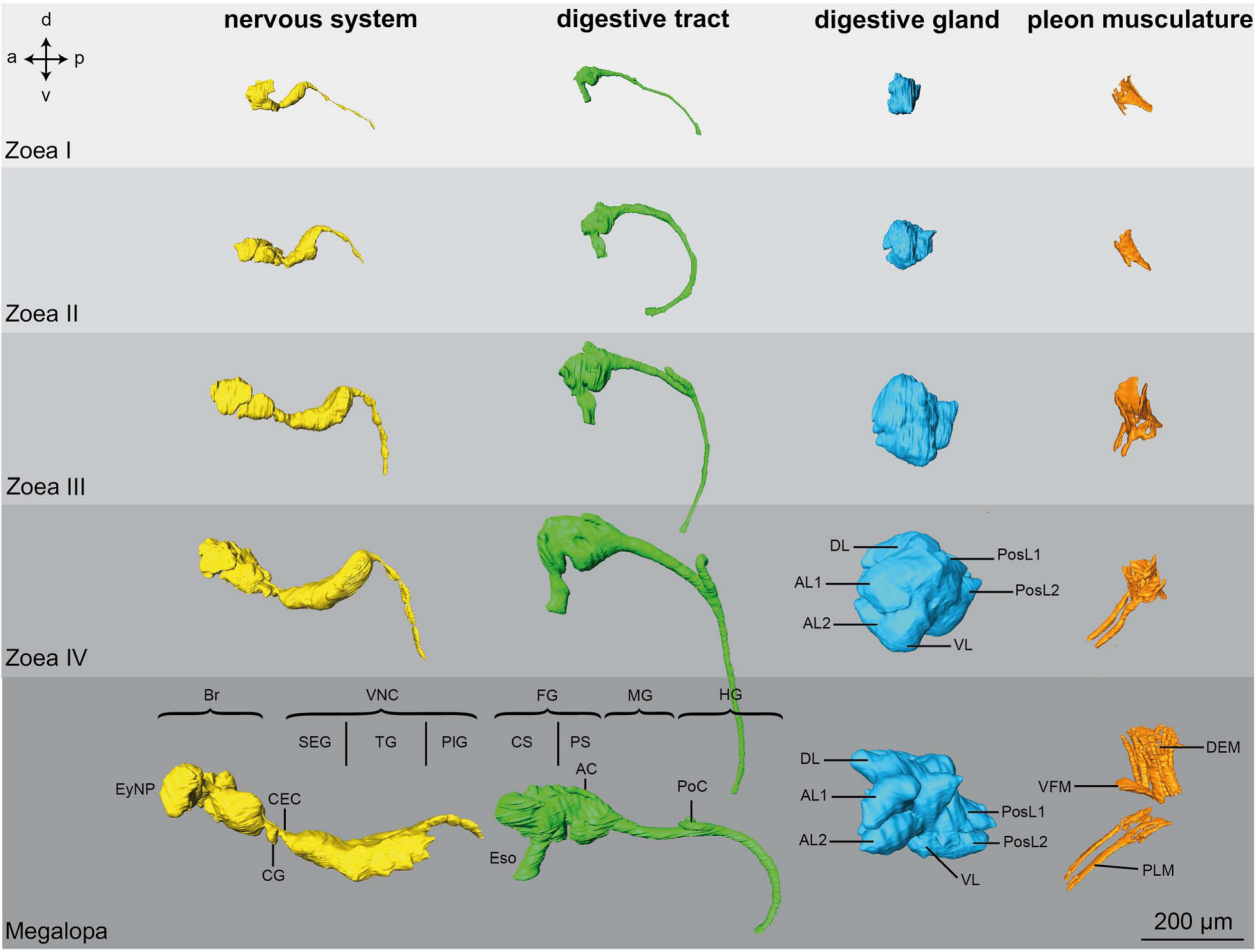
Ontogeny of selected organs based on micro-CT analyses, lateral views of 3D reconstructions. Crossed arrows indicate the orientation (a - anterior, d - dorsal, p - posterior, v - ventral). Abbreviations: AC - anterior caeca, AL1-2 - anterior lobe 1-2, Br – median brain, CEC - circumoesophageal connective, CG - commissural ganglion, CS - cardiac stomach, DEM - dorsal extensor muscles, DL - dorsal lobe, Eso - oesophagus, EyNP - eyestalk neuropil, FG - foregut, HG - hindgut, MG - midgut, PlG - pleonal ganglia, PLM – pleopod muscles, PoC - posterior caecum, PosL1-2 - posterior lobe 1-2, PS - pyloric stomach, SEG - suboesophageal ganglia, TG - thoracic ganglia, VFM - ventral flexor muscles, VL - ventral lobe, VNC - ventral nerve cord

**Fig. 5:**
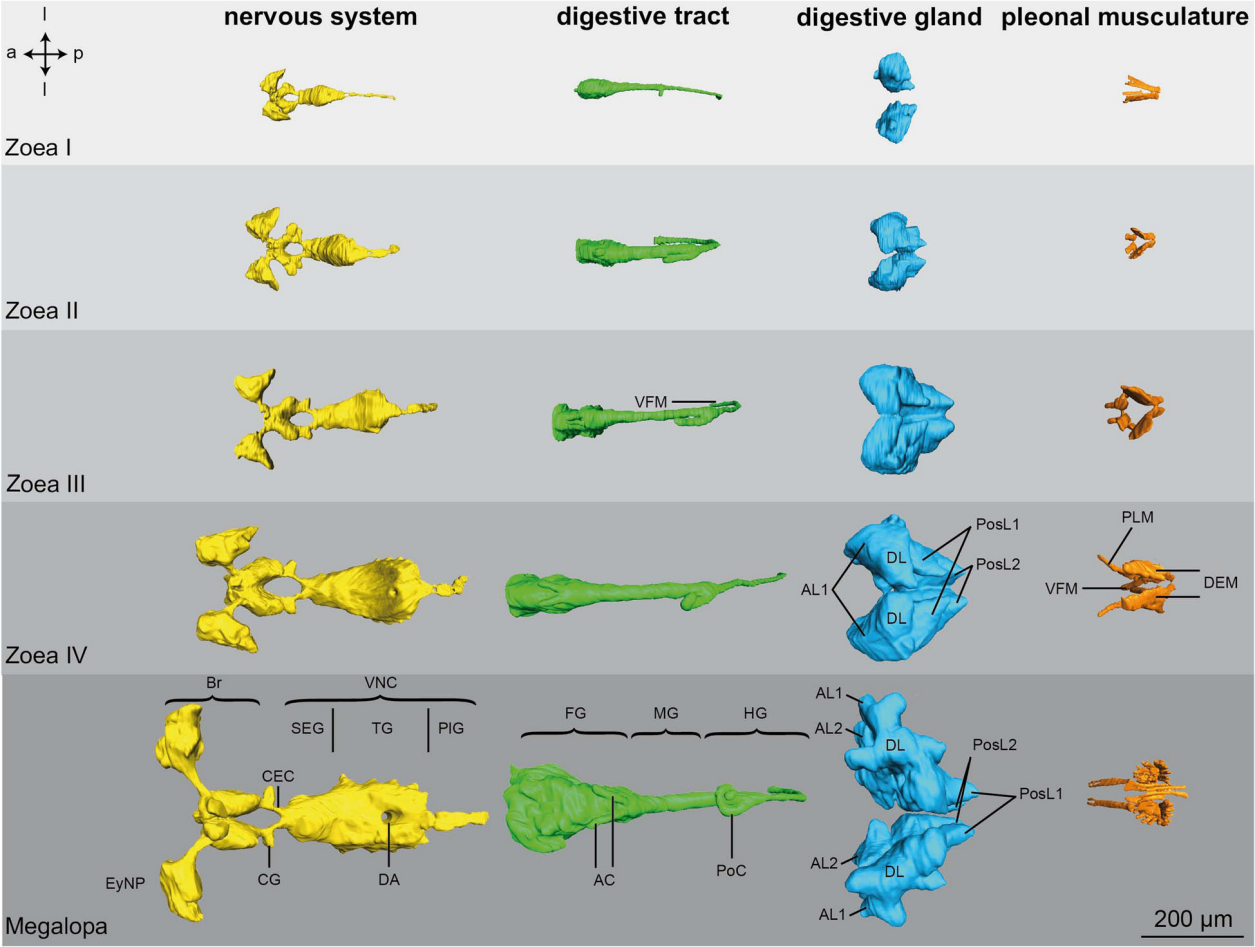
Development of selected organs from the Zoea I to the Megalopa based on micro-CT-analyses, dorsal views of 3D reconstructions. Crossed arrows indicate the orientation (a - anterior, l -lateral, p - posterior). Abbreviations: AC - anterior caeca, AL1, 2 - anterior lobes 1, 2, Br – median brain, CEC - circumoesophageal connective, CG - commissural ganglion, DA - descending artery, DEM - dorsal extensor muscles, DL - dorsal lobe, Eso - oesophagus, EyNP - eyestalk neuropil, FG - foregut, HG - hindgut, MG - midgut, PlG - pleonal ganglia, PoC - posterior caecum, PosL1-2 - posterior lobe 1-2, SEG - suboesophageal ganglia, TG - thoracic ganglia, VFM - ventral flexor muscles, VL - ventral lobe, VNC - ventral nerve cord

**Fig. 6:**
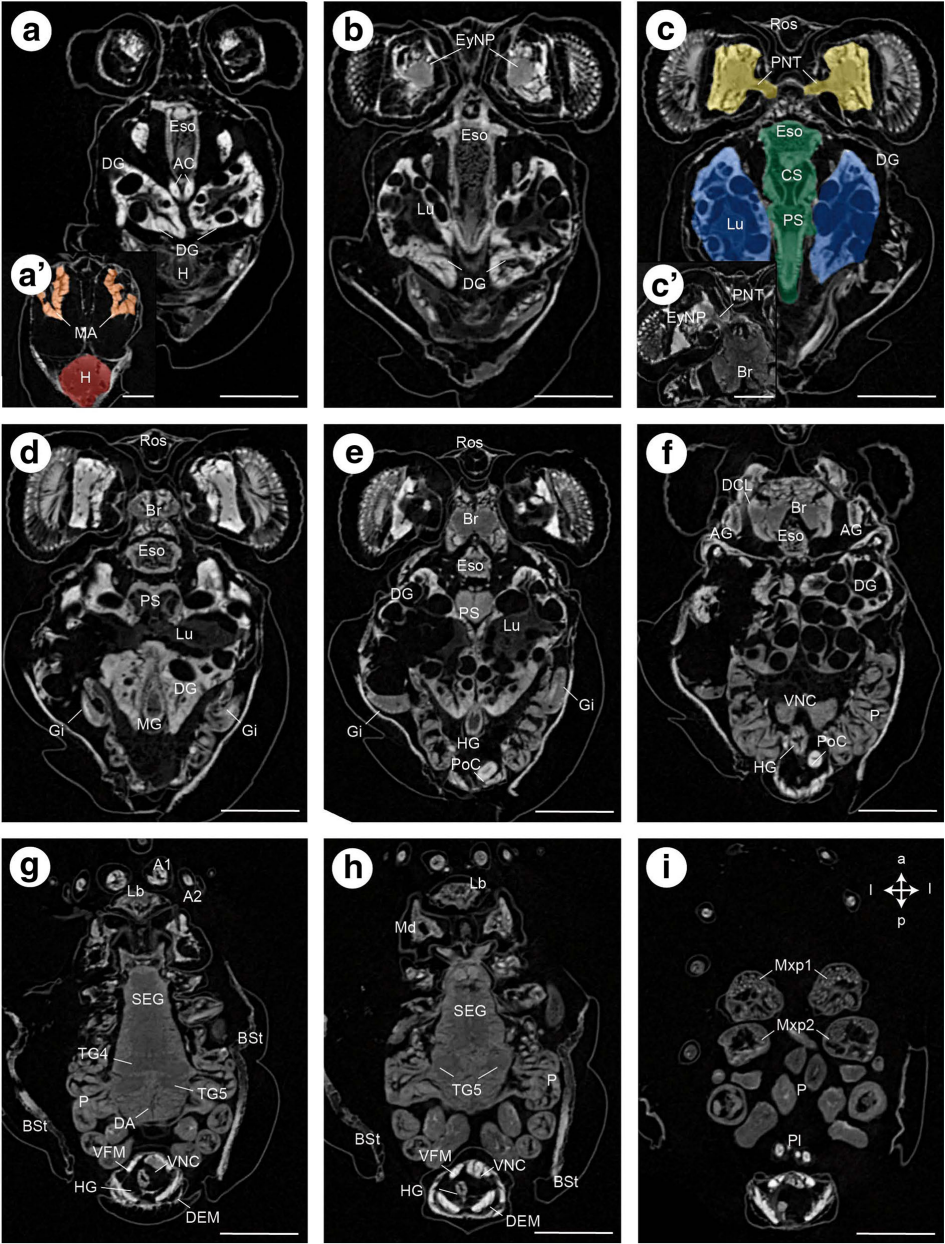
Zoea IV, consecutive horizontal sections from micro-CT analysis, from dorsal (**a**) to ventral (**i**). In **a** and **c**, the organs are coloured according to code used in Fig. 5. Crossed arrows in I indicate the orientation (a - anterior, l -lateral, p - posterior). Abbreviations: A1 - first antenna, A2 - second antenna, AC - anterior caecum, AG - antennal gland, Br – median brain, Bst - epithelium of the branchiostegite, CS - cardiac stomach, DA – descending artery, DCL - deutocerebral chemosensory lobe, DEM – dorsal extensor muscle, DG - digestive gland, Eso - oesophagus, EyNP - eyestalk neuropil, Gi - gill, H - heart, HG - hindgut, Lb - labrum, Lu - lumen, MA - mandibular adductor musculature, Md - mandible, Mxp1 - first maxilliped, Mxp2 - second maxilliped, PNT - projection neuron tract, P - pereiopod anlagen, Pl - pleopod anlagen, PoC - posterior caecum, PS - pyloric stomach, Ros - rostrum, SEG - suboesophageal ganglia, TG - thoracic ganglia, VFM - flexor muscles, VNC –ventral nerve cord. Scale bars represent 200 μm

**Fig. 7:**
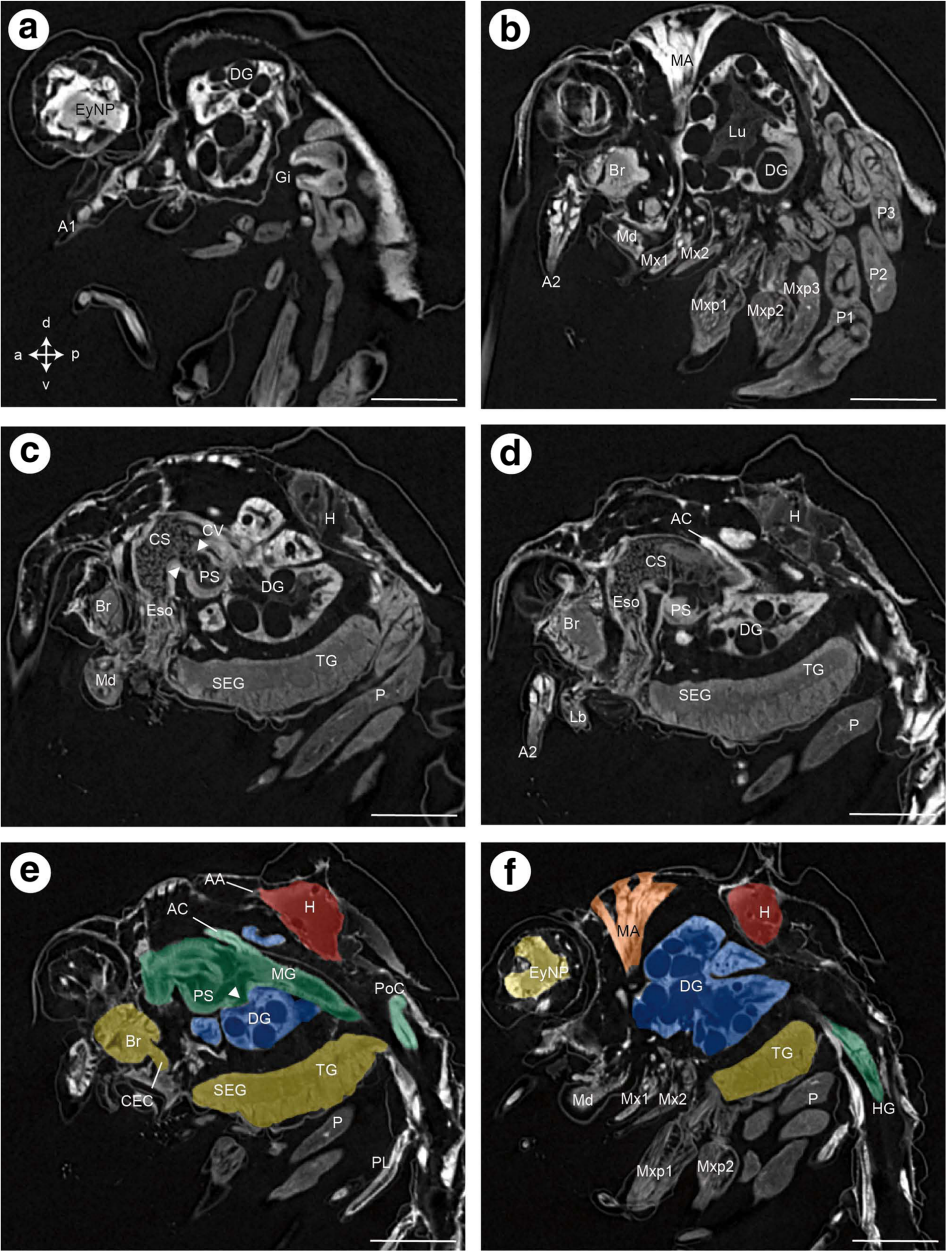
Zoea IV, consecutive lateral sections (**a** - **f**) from micro-CT analysis. In **e** and **f**, the organs are coloured according to code used in Fig. 5. Crossed arrows in A indicate the orientation (a - anterior, d – dorsal, p – posterior, v - ventral). Abbreviations: A1 - first antenna, A2 - second antenna, AC - anterior caecum, AG - antennal gland, Br – median brain, Bst - epithelium of the branchiostegite, CS - cardiac stomach, CV – cardio-pyloric valve, DA - descending artery, DCL - deutocerebral chemosensory lobe, DEM – dorsal extensor muscle, DG - digestive gland, Eso - oesophagus, EyNP - eyestalk neuropil, Gi - gill, H - heart, HG - hindgut, Lb - labrum, Lu – lumen of digestive gland, MA - mandibular adductor musculature, Md - mandible, Mxp1 - first maxilliped, Mxp2 - second maxilliped, Mxp3 - third maxilliped, PNT - projection neuron tract, P - pereiopod anlagen, Pl - pleopod anlagen, PoC - posterior caecum, PS - pyloric stomach, Ros - rostrum, SEG - suboesophageal ganglia, TG - thoracic ganglia, VFM - flexor muscles, VNC - ventral nerve cord. Scale bars represent 200 μm

**Fig. 8:**
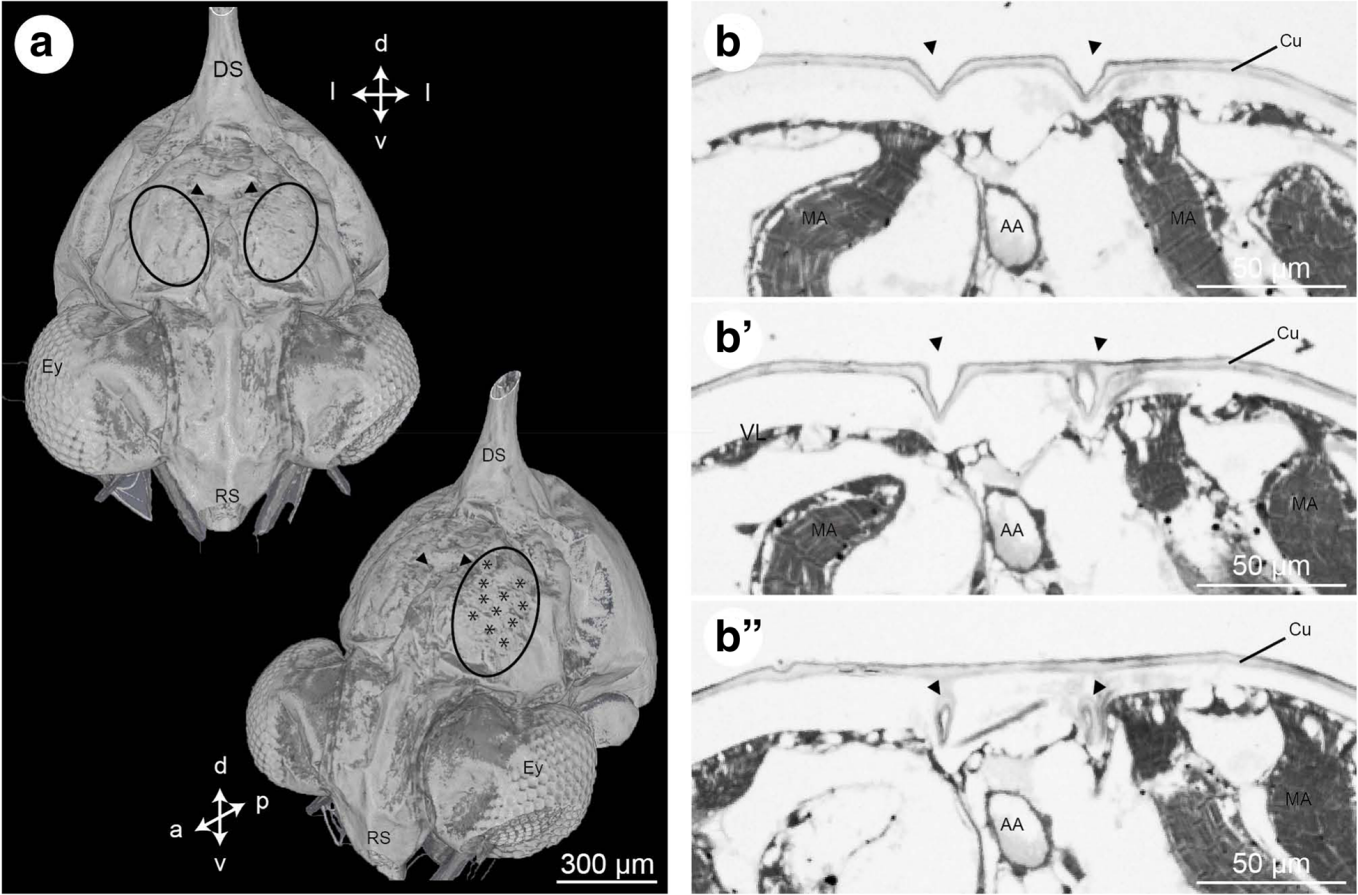
**a** - Zoea IV, isosurface of micro-CT-scans showing the external pores of the dorsal organ (black arrowheads) between the attachment sites of the mandibular adductor muscles (circles). Asterisks indicate the attachment of single muscle bundles. Crossed arrows indicate the orientation (a - anterior, d – dorsal, p – posterior, v - ventral). **b** – Zoea IV, consecutive frontal semi-thin sections through the pores (arrowheads) of the dorsal organ (anterior is towards the top). Abbreviations: AA - anterior aorta, Cu - cuticle, DS - dorsal spine, Ey - compound eye, MA - mandibular adductor musculature, RS - rostral spine

**Fig. 9:**
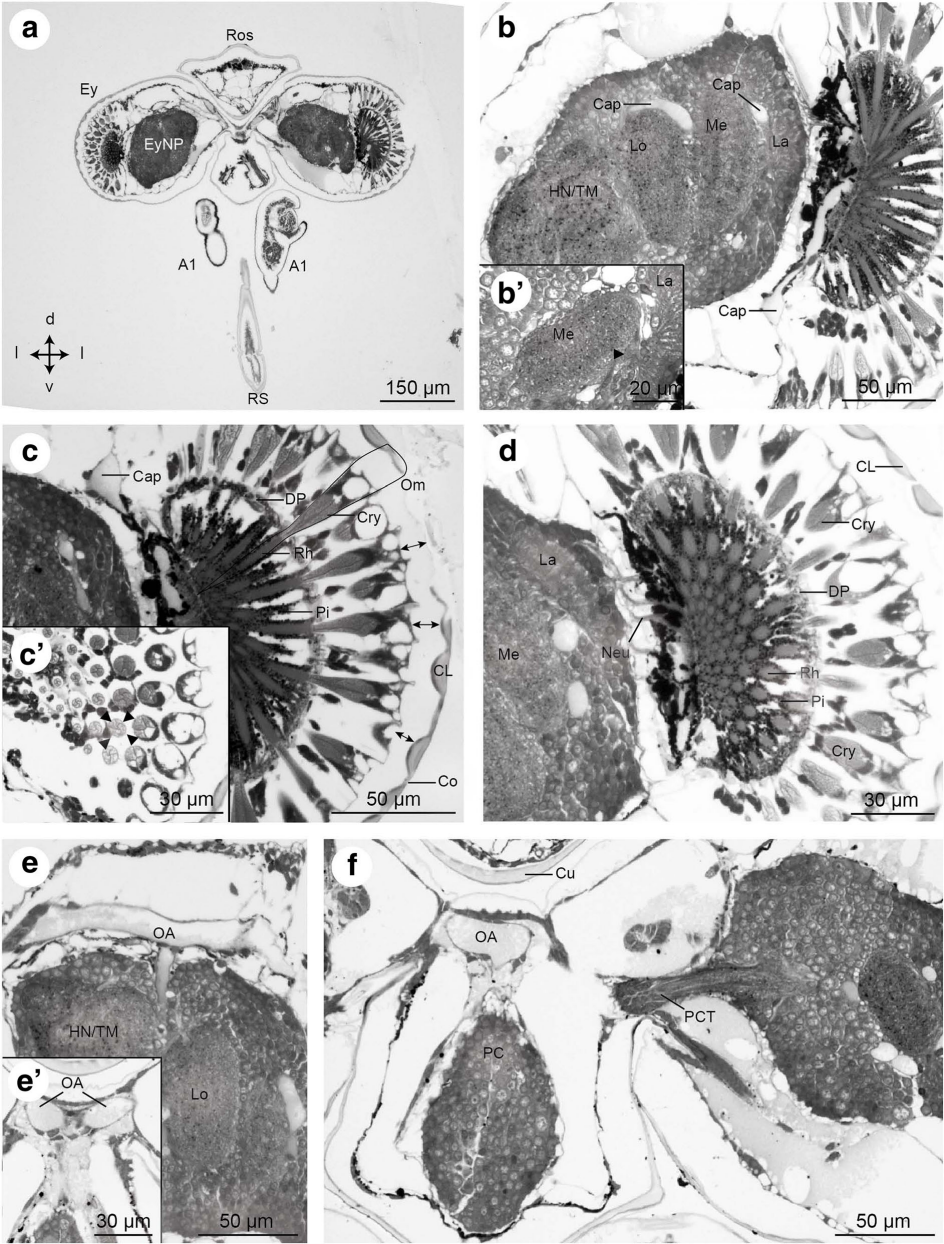
Frontal histological sections of Zoea IV (Holländer stain). **a** - overview of the anterior part of the head with stalked compound eyes and eyestalk neuropils (EyNP), see Fig. 2c for section plane; **b** - higher magnification of the right compound eye showing the visual neuropils lamina (La), medulla (Me), and lobula (Lo), proximally adjoined by the complex of the hemiellipsoid body neuropil and the medulla terminalis (HN/TM); b` - visual chiasm (arrow head) between medulla and lamina; **c** – the compound eye consists of many single ommatidia (one ommatidium surrounded by a line), note that the cornea is detached because of poor fixation (↔ double arrow); c`- cross sections of ommatidia show that the crystalline cones are composed of four cone cells. Each cell of one crystalline cone is indicated by an arrowhead; **d** - the ommatidia extend bundles of neurites (Neu) into the lamina; **e** – a branch of the ophthalmic artery (OA) extends towards the eyestalk neuropils; e`- the two main branches of the ophthalmic artery (OA) course towards the eyestalks; **f** – the protocerebral tract (PCT) connects the eyestalk neuropils and protocerebrum (PC) of median brain. Abbreviations: A1 - first antenna, Cap - capillary, CL - corneal lens, Co - cornea of compound eye, Cry - crystalline cone, Cu - cuticle, DP - distal pigment, Ey - compound eye, EyNP - eyestalk neuropils, HN/TM - complex of hemiellipsoid body neuropil and medulla terminalis, La - lamina, Lo - lobula, Me - medulla, Neu - neurites, OA - ophthalmic artery, Om - ommatidium of compound eye, PC - protocerebrum, PCT - protocerebral tract, Pi - shielding pigment, Rh - rhabdomeres of photoreceptor cell, Ros - rostrum, RS - rostral spine

**Fig. 10:**
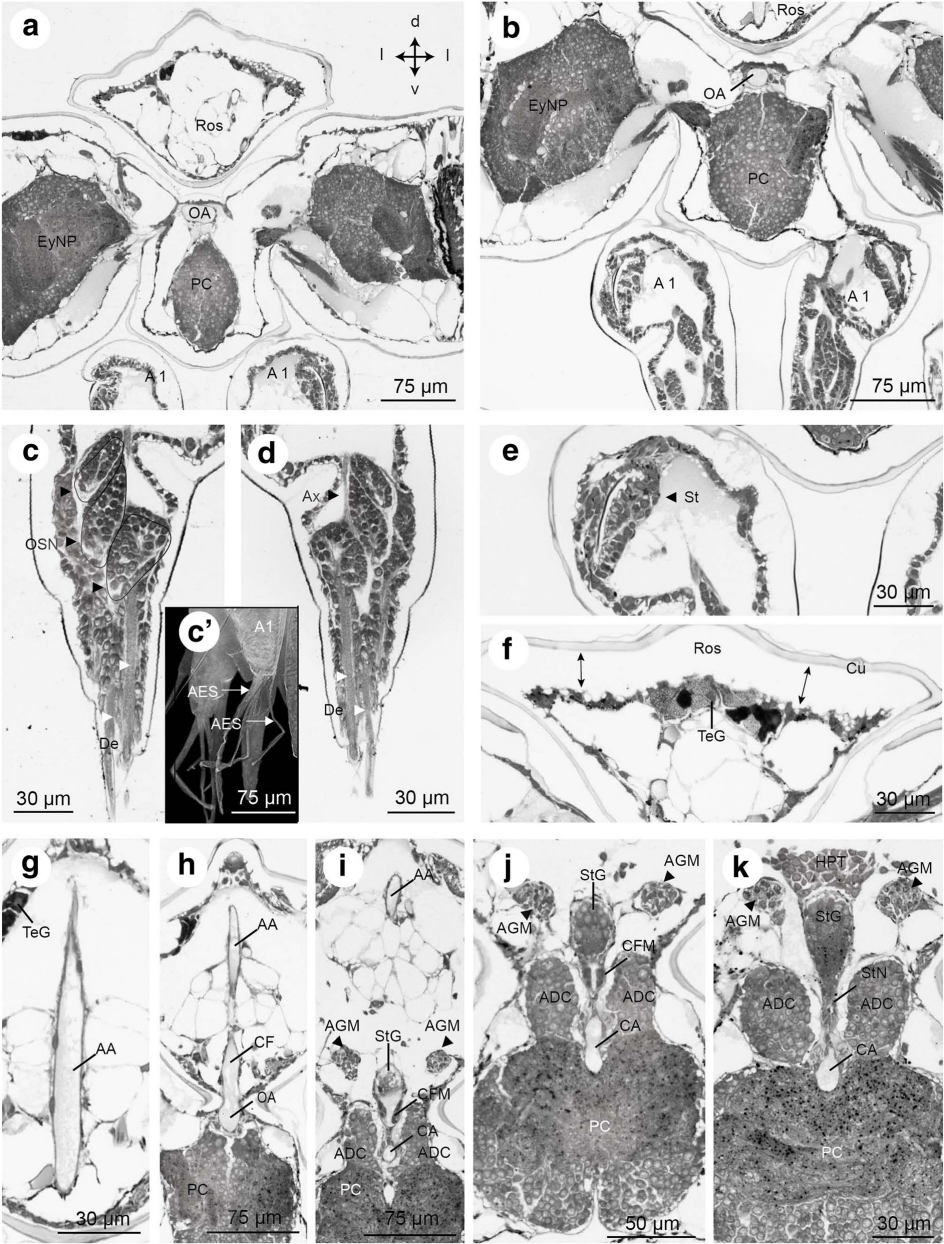
Frontal histological sections of Zoea IV (Holländer stain). **a** - overview of the anterior part of the head showing the eyestalk neuropils (EyNP), and medially the most anterior part of the protocerebrum (PC), see Fig. 2C for section plane; **b** - section slightly posterior to A showing the ophthalmic artery (OA) dorsal to the protocerebrum (PC); **c** - clusters of olfactory sensory neurons (OSN, circled and labelled with black arrowheads) within the first antenna send bundles of dendrites (De, white arrowheads) into aesthetasc sensilla (AES); c`- autofluorescence image of first antenna (A1) with aesthetascs (AES); **d** - axons (Ax) of olfactory sensory neurons extend towards the median brain; **e** – putative anlage of the statocyst (St) at the base of the first antenna; **f** - tegumental gland (TeG) on the dorsal side of the rostrum (Ros); note that cuticle (Cu) and epidermis are detached (double arrow); **g** - anterior aorta (AA) in the rostrum (Ros); **h** – the anterior aorta (AA) opens into to the auxiliary heart, the cor frontale (CF), from which the ophthalmic artery (OA) emerges; **i** – the cephalic artery (CA) emerges from the cor frontale. The anterior dorsal cells (ADC) are located dorsal to the protocerebral neuropil. The stomatogastric ganglion (StG) within the cor frontale is flanked by the anterior gastric muscles (AGM); **j** – higher magnification slightly posteriorly than I showing the cor frontale muscles (CFM) ventral to the stomatogastric ganglion (StG); **k** – the cephalic artery (CA) is flanked by the anterior dorsal cells (ADC) of the protocerebrum as it penetrates into the protocerebrum. The stomatogastric nerve (StN) connects the stomatogastric ganglion to the protocerebrum (PC). The anterior part of the hematopoietic tissue (HPT) is located dorsally to the cor frontale that embraces the stomatogastric ganglion. Abbreviations: A1 - first antenna, AA - anterior aorta, ADC - anterior dorsal cells of the protocerebrum, AES - aesthetasc, AGM - anterior gastric muscle, Ax - axon, CA - cephalic artery, Cap - capillary, CF - cor frontale, CFM - cor frontale muscle, Cu - cuticle, De - dendrites, EyNP - eyestalk neuropils, HPT - hematopoietic tissue, OA - ophthalmic artery, OSN - olfactory sensory neurons, PC - protocerebrum, Ros - rostrum, St – putative statocyst anlage, StG - stomatogastric ganglion, StN - stomatogastric nerve, TeG - tegumental gland

**Fig. 11:**
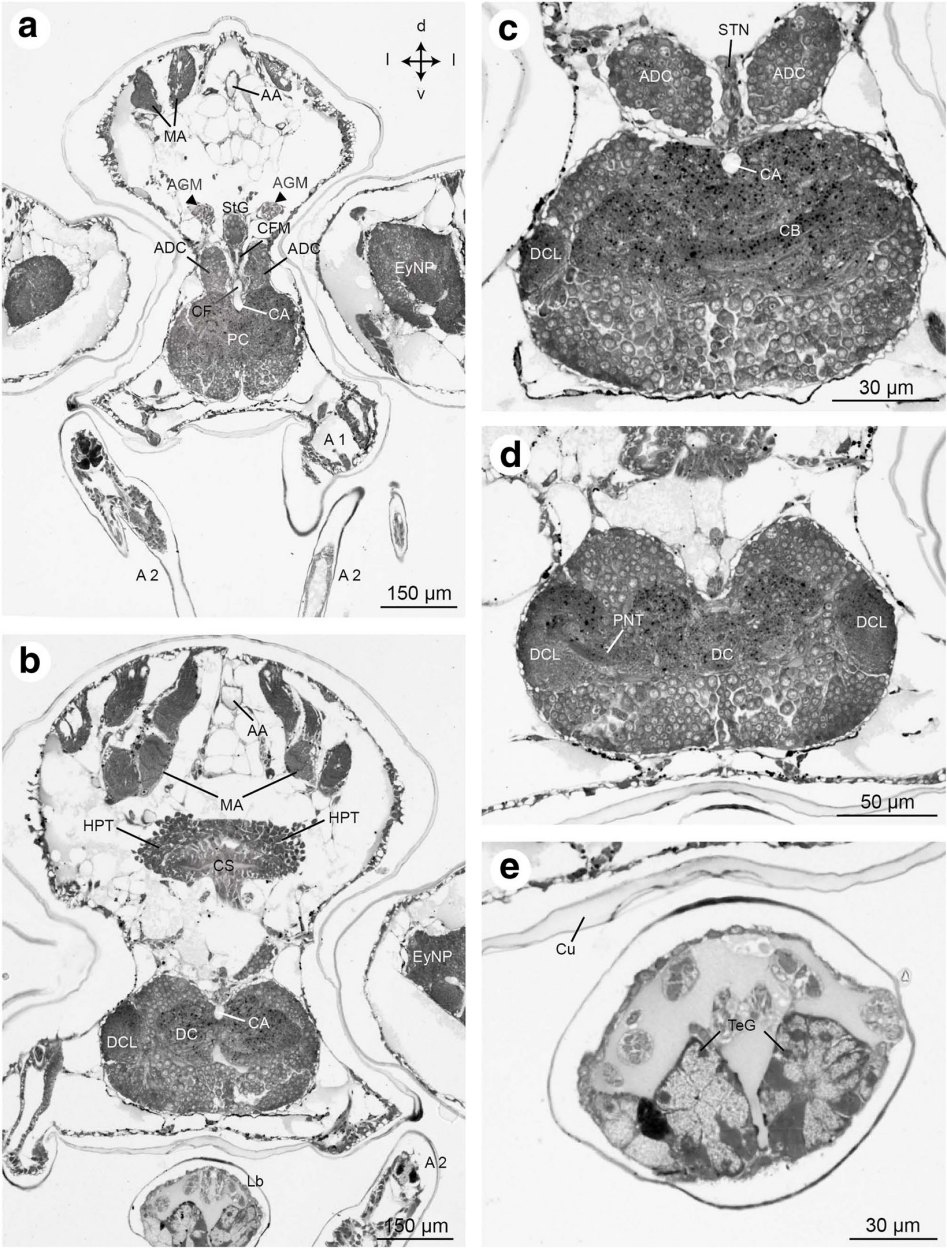
Frontal histological sections of Zoea IV (Holländer stain). **a** - overview showing the median brain and anterior part of the cephalothorax, see Fig. 2C for section plane; **b** - overview slightly posterior to A showing the prominent mandibular adductor musculature (MA) dorsally, on the ventral side the labrum (Lb) and second antenna (A2). Hematopoietic tissue is associated dorsally and laterally with the wall of the cardiac stomach (CS); **c** – the unpaired central body (CB) is located in the centre of the protocerebrum that is dorsally surmounted by the anterior dorsal cells (ADC). The stomatogastric nerve (STN) is also visible. **d** – the deutocerebrum is characterized by spherical deutocerebral chemosensory lobes (DCL) on both sides. The projection neuron tract (PNT) emerges from the left DCL. **e** – rosette-shaped tegumental gland (TeG) in the labrum. Abbreviations: A1 - first antenna, A2 - second antenna, AA - anterior aorta, ADC - anterior dorsal cells of the protocerebrum, AGM - anterior gastric muscle, CA - cephalic artery, CB - central body, CF - cor frontale, CFM - cor frontale muscle, CS - cardiac stomach, Cu - cuticle, DC - deutocerebrum, DCL - deutocerebral chemosensory lobe, EyNP - eyestalk neuropil, HPT - hematopoietic tissue, Lb - labrum, MA - mandibular adductor musculature, PC - protocerebrum, PNT - projection neuron tract, Ros - rostrum, StG - stomatogastric ganglion, StN - stomatogastric nerve, TeG - tegumental gland

**Fig. 12:**
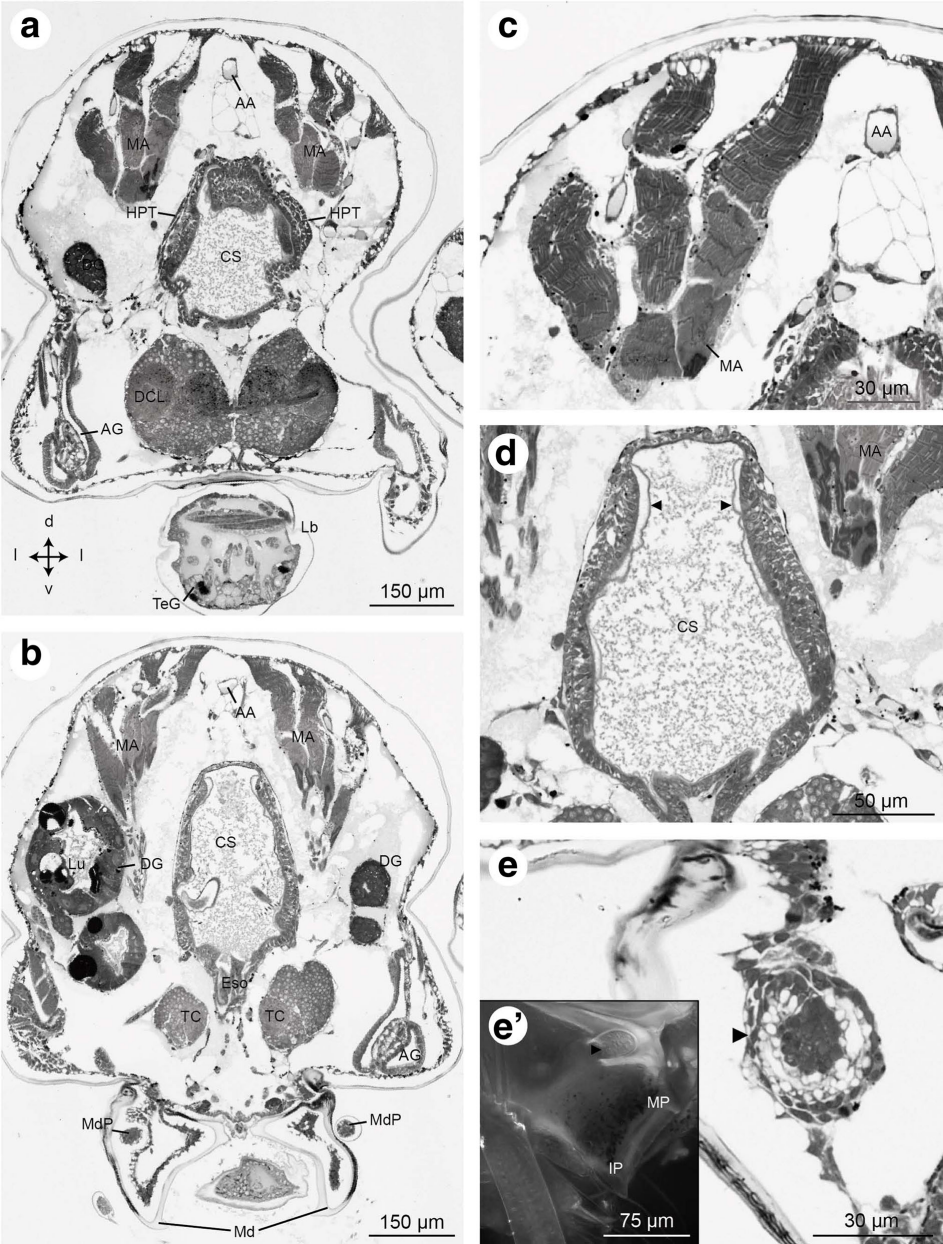
Frontal histological sections of Zoea IV (Holländer stain). **a** - section showing the median brain at the level of the deutocerebral chemosensory lobes and the cardiac stomach, see Fig. 2C for section plane; **b** - section slightly more posterior to A showing the tritocerebrum (TC) flanking the oesophagus (Eso), and the anterior lobes of the digestive gland with its lumen (Lu) and large lipid inclusions (black dots). The right antennal gland (AG) is also visible. **c** - mandibular adductor musculature (MA) displays the characteristic cross striation; **d** - the anterior part of the cardiac stomach (CS) inside is lined with cuticle (arrowheads); **e** - inner portion of the anlage of the mandibular palp located inside of the mandible; e`- autofluorescence of the mandible with the outer portion of the mandibular palp anlage (arrowhead). Abbreviations: AA - anterior aorta, AG - antennal gland, CS - cardiac stomach, DCL - deutocerebral chemosensory lobe, DG - digestive gland, Eso - oesophagus, HPT - hematopoietic tissue, Lb - labrum, Lu – lumen of digestive gland, MA - mandibular adductor musculature, Md - mandible, MdP – mandibular palp, TC - tritocerebrum, TeG - tegumental gland

**Fig. 13:**
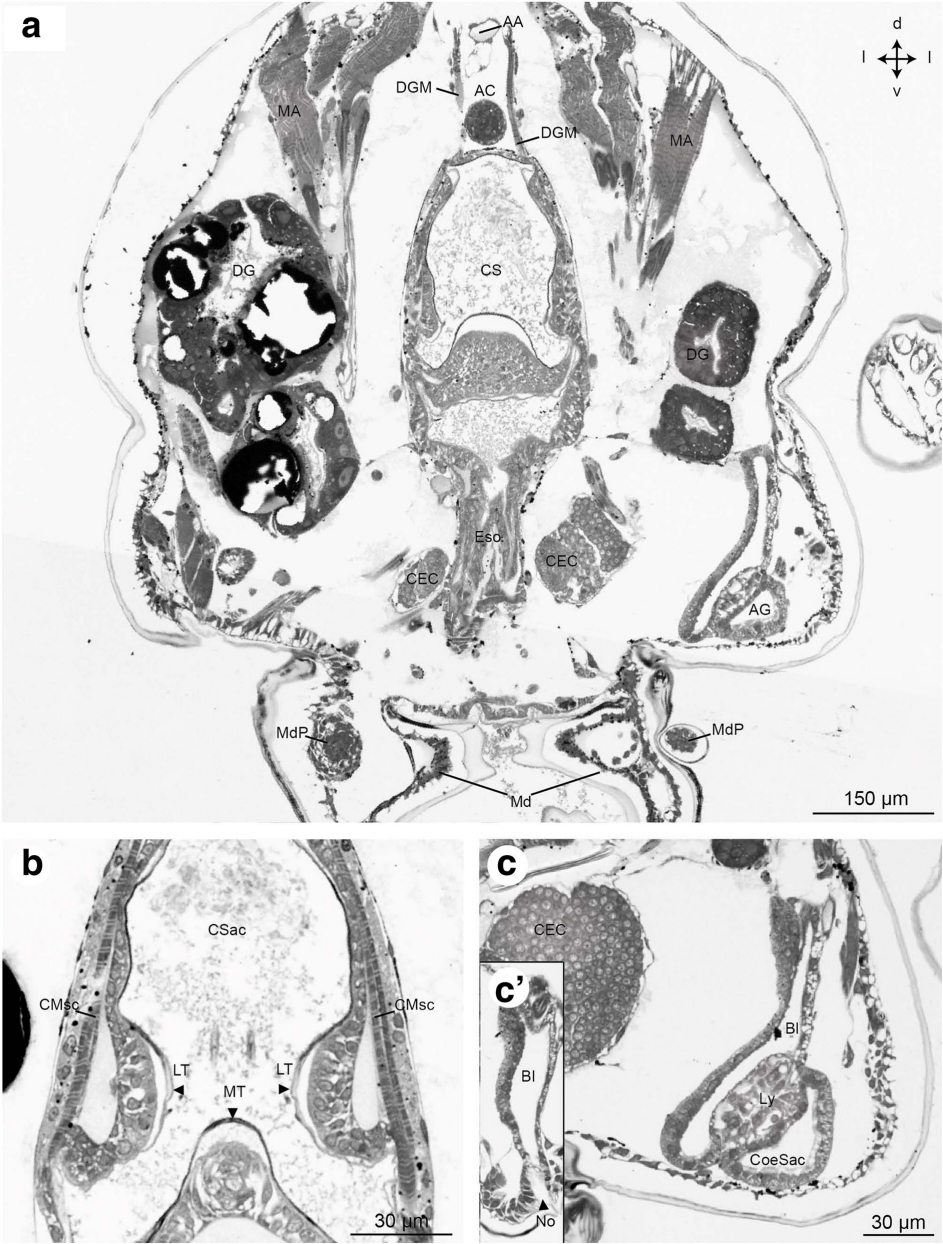
Frontal histological sections of Zoea IV (Holländer stain). **a** - section through the median part of the cardiac stomach (CS), see Fig. 2c for section plane; **b** - higher magnification of the cardiac stomach with the gastric mill that comprises two lateral teeth (LT) and one median tooth (MT), note the thickened cuticle of the teeth as indicated by black arrowheads; **c** - higher magnification of the antennal gland; c` - the bladder (BL) of the antennal gland opens to the outside through a nephropore (No). Abbreviations: AA - anterior aorta, AC - anterior caecum, AG - antennal gland, Bl - bladder, CEC - circumoesophageal connective, CMsc - contractor muscles of the cardiac stomach, CoeSac - coelomosac, CS - cardiac stomach, CSac - cardiac sac, DG - digestive gland, DGM - dorsal gastric muscles, Eso - oesophagus, LT - lateral tooth, Ly - labyrinth, MA - mandibular adductor musculature, Md - mandible, MdP – mandibular palp, MT - median tooth, No - nephropore, TC – tritocerebrum

**Fig. 14:**
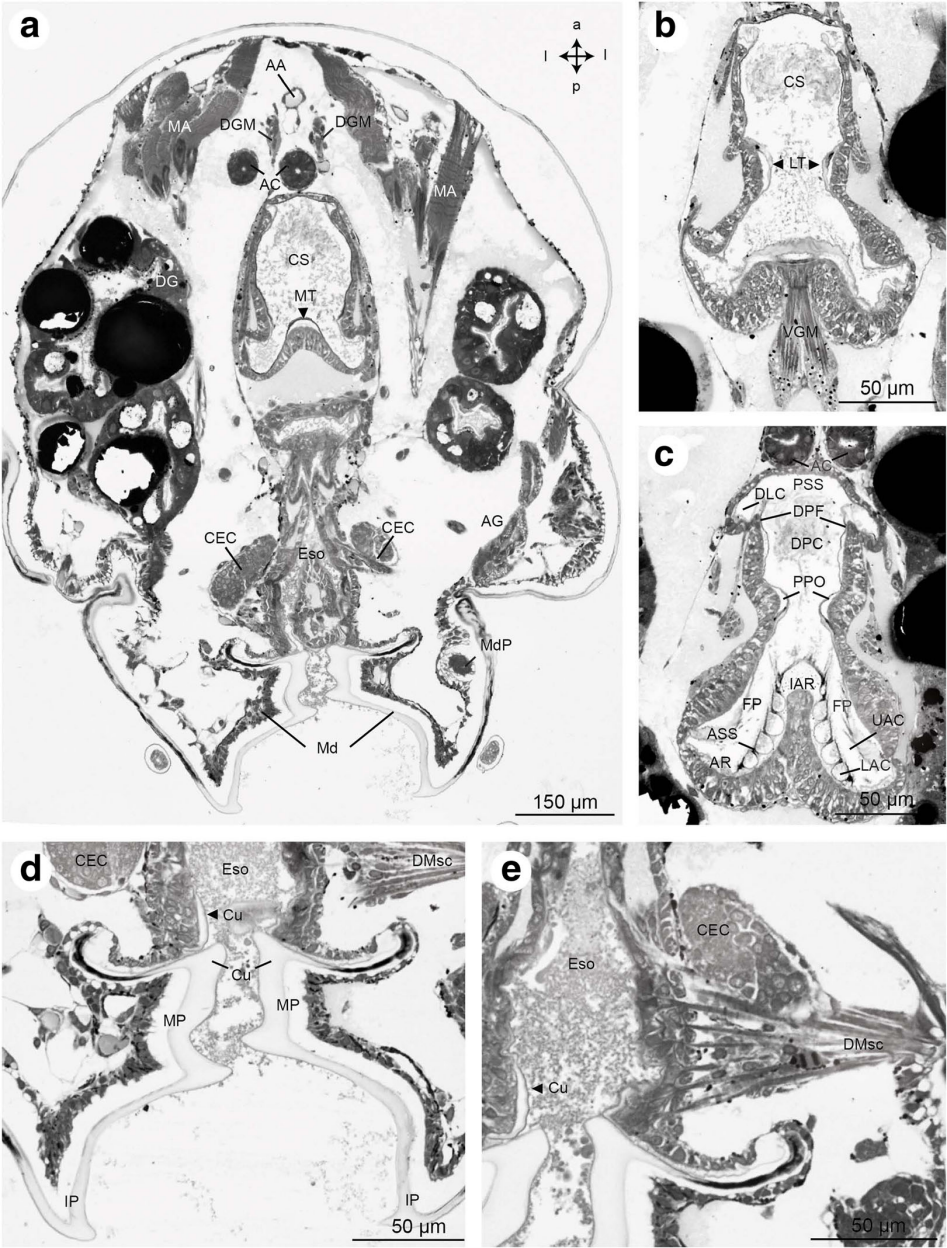
Frontal histological sections of Zoea IV (Holländer stain). **a** - section of the cephalothorax with the cardiac stomach (CS) in the centre. Dorsally, the paired anterior caeca (AC) are located. Ventrally the oesophagus (Eso) opens to the mouth field, laterally flanked by the mandibles (Md); compare Fig. 2c for section plane; **b** - posterior part of the cardiac stomach with sclerotized lateral teeth (LT). Ventrally, the ventral gastric muscles are attached (VGM). **c** - details of the pyloric stomach with gastric mill. **d** - higher magnification of the sclerotized mandible (Md), note the massive cuticle (Cu) of the molar process (MP); **e** - detail of the dilatator muscles (DMsc) attached to the oesophagus (Eso). Abbreviations: AA - anterior aorta, AC - anterior caecum, AG - antennal gland, AR - ampullary ridge, ASS - ampullary setal screen of the gland filter, CEC - circumoesophageal connective, CMsc - dilatator muscles of the cardiac stomach, CS - cardiac stomach, Cu - cuticle, DG - digestive gland, DGM - dorsal gastric muscles, DLC - dorsolateral pyloric channel, DMsc - dilatator muscles of the oesophagus, DPC - dorsal region of the pyloric chamber, DPF - dorsal pyloric fold, Eso - oesophagus, FP - filter press, IAR - interampullary ridge, IP – incisor process, LAC - lower ampullary chamber, MA - mandibular adductor musculature, Md - mandible, MdP – mandibular palp, MP – molar process, MT - median tooth, PPO - prepyloric ossicle, PSS - pyloric setal screen, UAC - upper ampullary chamber, VGM – ventral gastric muscle

**Fig. 15:**
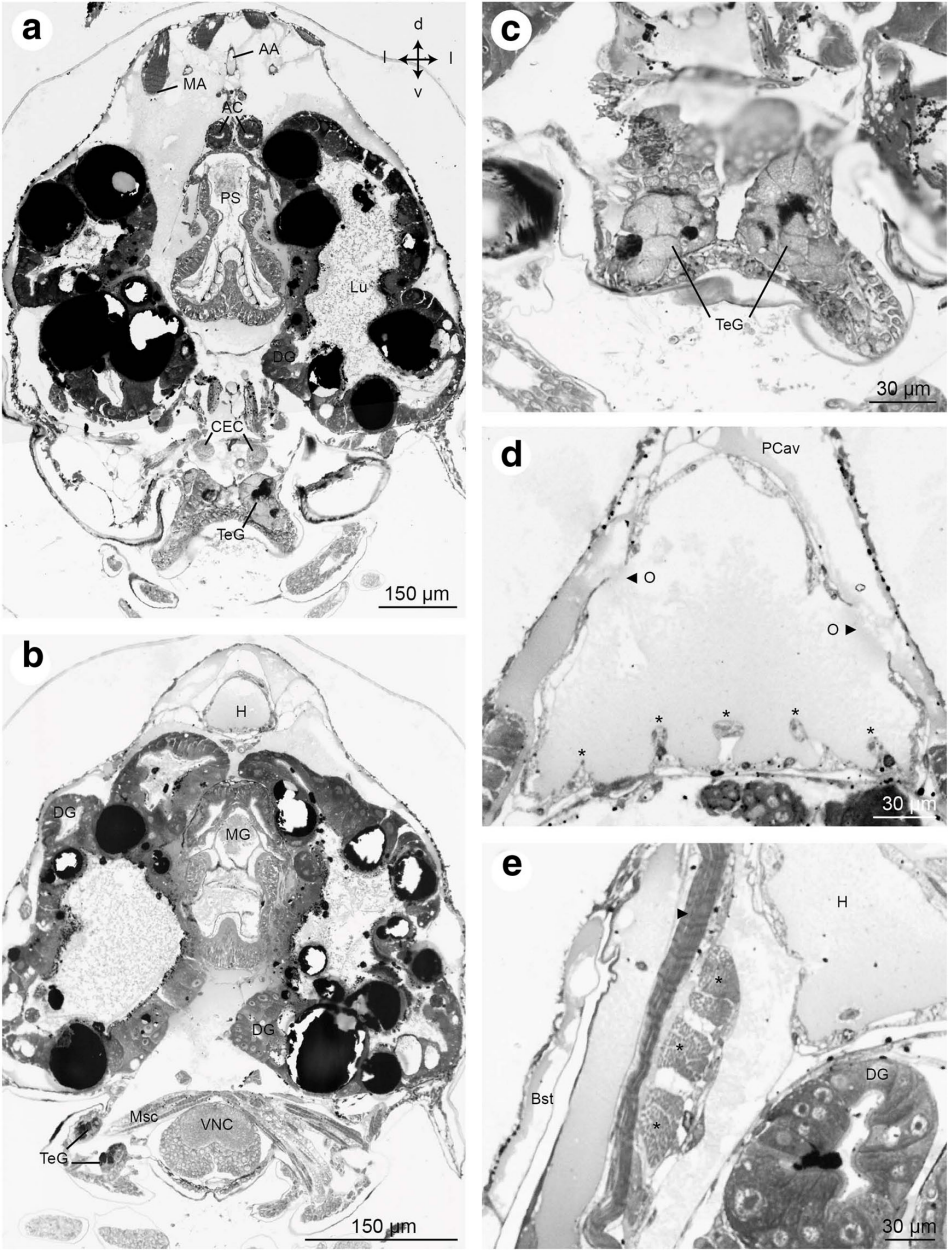
Frontal histological sections of Zoea IV (Holländer stain). **a** - section showing the median portion of the pyloric stomach (PS) laterally flanked by the lobes of the midgut gland, see Fig. 2c for section plane; **b** - section of the anterior part of the midgut (MG) with the dorsal heart (H) and the ventral nerve cord (VNC) at the level of the suboesophageal ganglia. Dorsally, the VNC is crossed by muscles (Msc) associated with the maxillae; **c** - higher magnification of rosette-shaped tegumental glands of the mandible; **d** - the heart is surrounded by the pericardial cavity (PCav) into which the ostia (O) open. Asterisks identify putative trabeculae; E - cross section of musculature (asterisks and arrow) laterally to the heart (H) underneath the branchiostegites (Bst). Abbreviations: AA - anterior aorta, AC - anterior caecum, CEC - circumoesophageal connective, DG - digestive gland, H - heart, Lu – lumen of digestive gland, MA - mandibular adductor musculature, MG - midgut, Msc - musculature of the maxillae, PS - pyloric stomach, TeG - tegumental gland, VNC - ventral nerve cord

**Fig. 16:**
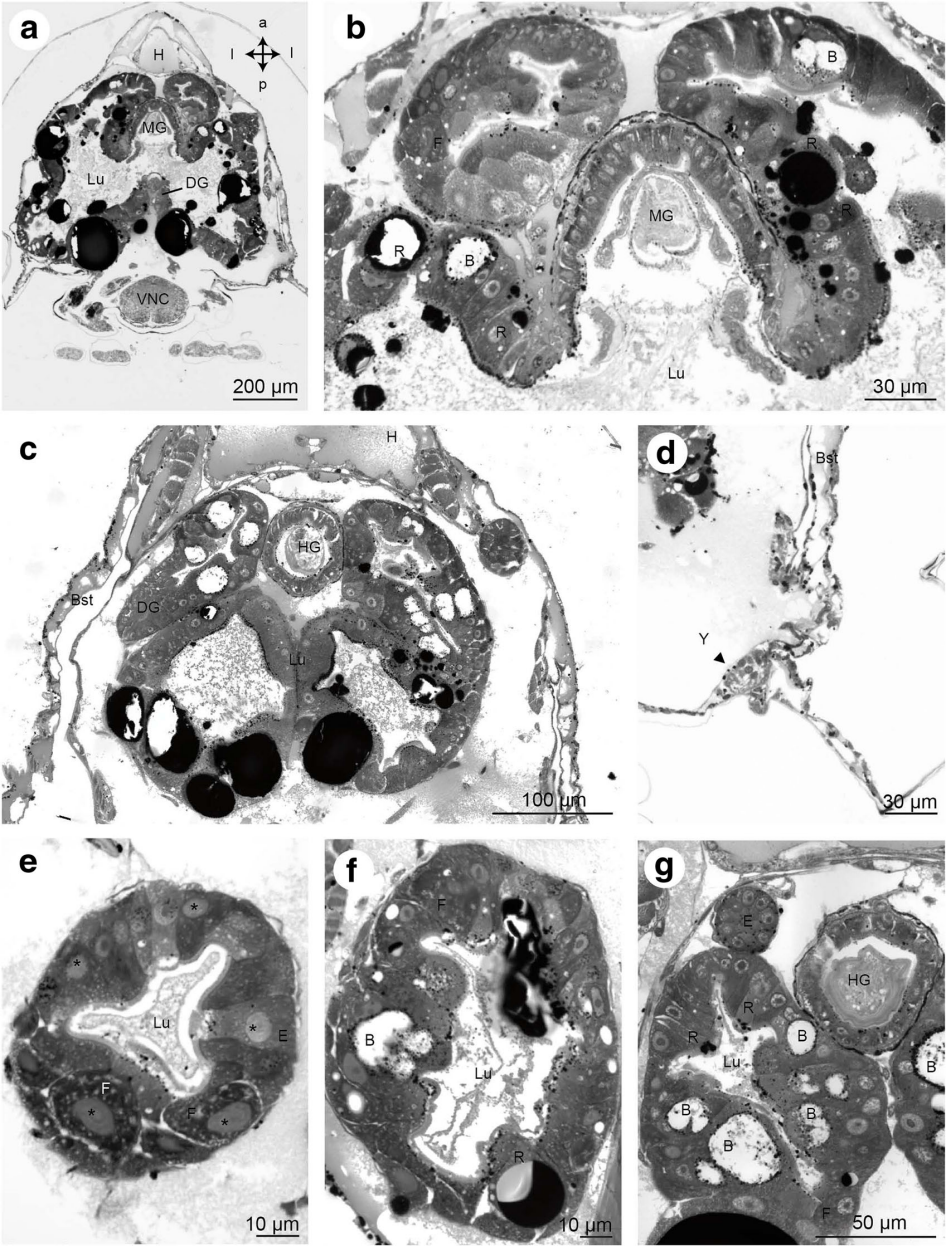
Frontal histological sections of Zoea IV (Holländer stain). **a** - cross-section showing the lobes of the digestive gland (DG) opening into the midgut (MG), see Fig. 2c for section plane; **b** - close-up of the interface of the digestive gland to the midgut (MG). Cell-types of the digestive gland identified include embryonic cells (E), blister like cells (B) and resorptive cells (R). **c** - section across the posterior lobes of the midgut gland and the anterior part of the hindgut (HG); **d** - the Y-organ (Y) is located underneath the lateral carapace close to the interface with the branchiostegites (Bst); **e** - distal part of a digestive gland lobe showing lumen (Lu) surrounded by epithelium composed of E- and F-cells. **f** - section through a lobe of the digestive gland showing epithelium with R- and B-cells surrounding the lumen (Lu). **g** - section of the digestive gland more posteriorly than A, the B-, F-, R-, and E-cells are identified. Abbreviations: B – blister like cell, Bst - epithelium of branchiostegite, DG - digestive gland, E - embryonic cell, F - fibrillary cell, H - heart, HG - hindgut, Lu – lumen of digestive gland, MG - midgut, R - resorptive cell, VNC - ventral nerve cord, Y - Y-organ

**Fig. 17:**
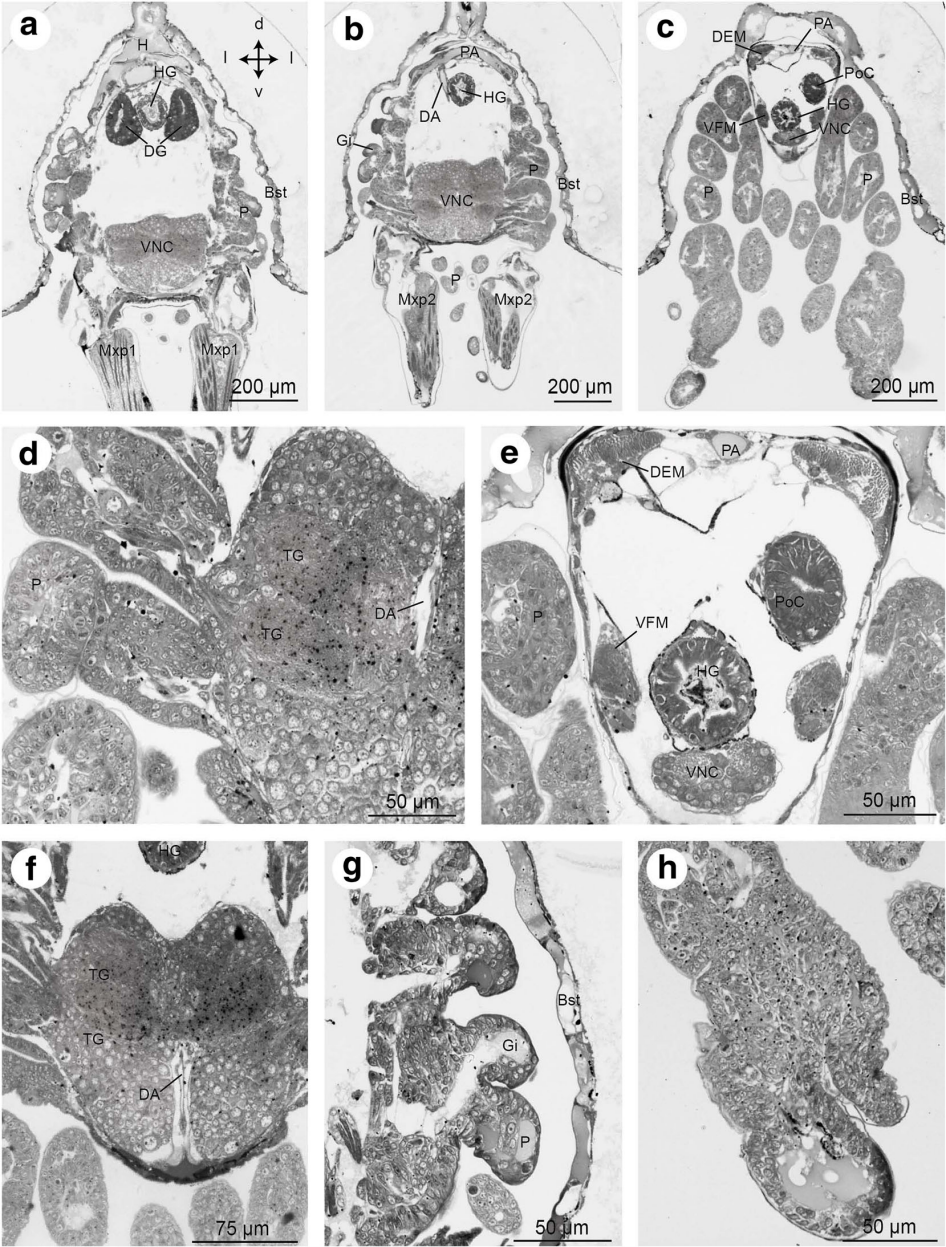
Frontal histological sections of Zoea IV (Holländer stain). **a** - section through the distal part of the digestive gland (DG) with the two posterior lobes flanking the hindgut (HG), see Fig. 2c for section plane. **b** - section showing the posterior artery (PA) as well as the descending artery (DA). **c** - section through the first pleon segment with the hindgut (HG) and its blindly ending posterior caecum (PoC) as well as pereiopod anlagen (P). **d** - higher magnification of the ventral nerve cord (VNC), penetrated by the descending artery (DA). **e** - higher magnification of the first pleon segment shown in B, the flexor muscles (VFM) and extensor muscles (DEM) of the pleon are identified. **f** - higher magnification of the ventral nerve cord (VNC) as it is penetrated by the descending artery (DA). **g** - undifferentiated tissue of pereiopod (P) and gill anlagen (Gi) surrounded by the lateral branchiostegites; **h** - close-up of undifferentiated tissue of a pereiopod anlage. Abbreviations: Bst - epithelium of branchiostegite, DA - descending artery, DEM - extensor muscle, DG - digestive gland, Gi - gill, H - heart, HG - hindgut, Mxp1 - first maxilliped, Mxp2 - second maxilliped, P - pereiopod anlagen 1 - 5, PA - posterior artery, PoC - posterior caecum, TG – thoracic ganglia, VFM - flexor muscle, VNC - ventral nerve cord

As in adult Brachyura, the digestive tract of all larval stages of *C. maenas* consists of three sections (green in Figs. 3, 4 and 5): the oesophagus (Eso) that connects to the two-chambered foregut (FG; also called proventriculus), the midgut (MG), and the hindgut (HG). The function of mechanically grinding the food is shared between the mandibles and the gastric mill of the foregut whereas digestion and absorption primarily occur within the midgut. In the following, we will describe the elemts of the degestive system based on the histological section series of a Zoea IV (Figs. 6, 7, 8, 9, 10, 11, 12, 13, 14, 15, 16 and 17).

#### Mandibles and foregut in the Zoea IV

The larval mouth is flanked by the pair of mandibles (Md, Fig. 14a). In frontal views of the Zoea IV, the molar process (MP) with its massive cuticle (Cu) and incisor process (IP) of the mandible can be distinguished (Fig. 14d). The anlage of the mandibular palp (MdP) has its origin in a dense, spherical accumulation of cells within the mandible from where this structure projects posteriorly and extends in parallel to the mandible (Fig. 12b, e, e’, 13). The tubular oesophagus (Eso) is composed of a multicellular epithelium and extends dorsally from the mouth (Figs. 13a, 14a, e). The inner side of the oesophagus is lined with a thin layer of cuticle showing its ectodermal origin (Fig. 14e). Distinct bundles of dilatator muscles (DMsc; Fig. 14d, e) are laterally attached to the oesophagus just dorsal to the mouth opening, possibly mediating peristaltic movements of the oesophagus. The oesophagus opens dorsally into the cardiac stomach (CS; also called anterior proventriculus) which forms the large anterior part of the foregut (Figs. 4, 7c, 12b, 13a). The cardiac stomach comprises the large cardiac sac (CSac), and the gastric mill composed of two lateral and one median teeth covered with thick cuticle (Fig. 13b). It is laterally flanked by distinct stands of striated muscle (CMsc, Fig. 13b) that may serve to contract the cardiac stomach. Paired muscle strands suspend the foregut to the integument dorsally (dorsal gastric muscles: DGM, Fig. 13a), anteriorly (anterior gastric muscles: AGM, Figs. 10i, 11a), and ventrally (ventral gastric muscles: VGM, Fig. 14b).

The cardiac stomach is separated from the adjoining, more ventrally arranged pyloric stomach (PS; Figs. 4, 6c, 7c, 15a; also called posterior proventriculus) by a slight constriction, the cardio-pyloric valve (CV; Fig. 7c). The pyloric stomach is composed of a dorsal region, the pyloric chamber (DPC) and a ventral region, the ampullary chamber (UAC and LAC; Fig. 14c). These chambers are separated from each other by a pair of prepyloric ossicles (PPO). The ventral ampullary chamber represents a complex filter system, the filter press (FP; also called gland filter; Figs. 14c, 17a), which prevents large particles from passing to the most ventral part of the pyloric chamber. The lateral sides of this ventral portion of the pyloric stomach are lined by setae. The median interampullary ridge (IAR) is also covered with ampullary setae (ASS) at its lateral sides. Liquid food components pass the filter press to reach the digestive gland.

#### Midgut and digestive gland in the Zoea IV

The midgut connects posteriorly to the dorsal region of the pyloric stomach (green in Fig. 7; see also Figs. 10e, 17b). At the junction between these two regions, the paired, anteriorly directed midgut caeca (AC) are attached dorsally (Figs. 4, 7e, 14a, 15a). The caeca end blindly and consist of a single layer of epithelial cells without visible specialization. The midgut itself is also composed of a single layer of columnar epithelial cells (Fig. 16b). The paired lobes of the digestive gland (DG) are connected to the ventral ampullary chamber and the filter press (Figs. 16a, b). Posteriorly to this interface with the digestive gland, the midgut is adjoined by the hindgut.

The digestive gland (also called midgut gland or hepatopancreas), is a paired organ system flanking and surrounding the foregut and midgut area, that occupies a major part of the cephalothorax (blue in Figs. 3, 4, 5, 6c, 7f). This organ is composed of an elaborate epithelium surrounding a lumen (Figs. 14a, 15a, b, 17a, c) that forms blindly ending lobes that extend anteriorly and posteriorly (blue in Figs. 4 and 5). On both sides of the organism, we could repeatedly distinguish several types of lobes in several specimens, one dorsal lobe (DL), one ventral lobe (VL), two anterior lobes (AL1, 2), and two posterior lobes (PosL1, 2; Figs. 4 and 5). The organ’s lumen has its widest extension in the median part of the gland (Fig. 16a). In adult brachyurans, its epithelium consists of at least four characteristic cell types: embryonic (E), fibrillary (F), resorptive (R), and blister like (B) cells (reviews [69, 70]). However, following the detailed cytological description laid out by Vogt [71, 50]) we traced poorly differentiated cells similar to E-cells located at the distal, blindly ending tips of the lobes (Fig. 16e, g). F-cells are also located at the distal end of the lobes and with our staining protocol could be differentiated from E-cells by the darker and more granular cytoplasm (Fig. 16e, f). The R-cells, contain numerous storage vesicles, which in our sections were visible as large, darkly stained inclusions that most likely include lipids (Fig. 16b, f; compare [72]). B-cells include large, unstained central areas (hence their name blister like; Fig. 16b f, g) surrounded by cytoplasm with accumulations of small, dark granula. Many of the cells in the midgut gland did not display such diagnostic features so that we were unable to identify them.

#### Hindgut in the Zoea IV

The hindgut is a simple, tubular structure that extends posteriorly throughout the entire pleon and ends in the anus. Like the midgut, it consists of an epithelium with a single layer of columnar cells (Fig. 17e). Another blindly ending caecum, the posterior caecum (poC), arises posteriorly from the hindgut, at the interface of the cephalothorax and the pleon (Fig. 17a-c, e). As for the anterior caeca, the epithelial cells of this structure are columnar and without further specification (Fig. 13c).

#### Ontogenetic changes

Based on micro-CT scans of all larval stages, we were able to reconstruct the gross anatomy of the digestive tract to visualize ontogenetic changes (Figs. 3, 4 and 5). Its principal components as described for the Zoea IV are present already at hatching. From the Zoea I to IV, there are gradual changes in its morphology. Also, according to our CT data the first metamorphic moult to the Megalopa is not accompanied by massive structural changes at the macroscopic level. Nevertheless, distinct growth in size and volume occurs from hatching onwards as larval development proceeds into the Megalopa. The midgut and hindgut including the caeca elongate concurrently with the general growth of the larvae (Fig. 4), and the cardiac and pyloric stomach increase in volume. Additionally, the morphological separation between the cardiac and pyloric stomach becomes more distinct towards the end of the larval development (Fig. 4). The volume of the midgut gland also increases massively and both the anteriorly and posteriorly protruding lobes become more and more distinctive and extend in length.

### Musculature

#### Musculature of the cephalothorax

From hatching onwards, a complex system of striated muscles drives the functional appendages from the mandible to the second maxillipeds. Among the most prominent muscles that can be distinguished already in the Zoea I (Fig. 3) are the paired mandibular adductor muscles (MA), that provide forceful movements of the mandibles, which in concert with the gastric mill macerate food items (for the Zoea IV, see Figs. 11a, b, 12c, 13a, 14a). These massive muscles are already visible in whole mount specimens under low power microscopic magnification (data not shown) and are attached dorsally to the integument of the cephalothorax just anteriorly of the dorsal spine (Figs. 3, 7b, 8). They taper ventrally where they attach to the mandible *via* a tendon. Each mandible adductor muscle consists of several bundles organized in three rows. From the Zoea I to the Zoea IV, a massive growth of these muscles is evident, concurrent with the growth of the entire organism (Fig. 3).

Spanning dorsally across the ventral nerve cord, bundles of medially fused muscles connect the proximal elements of the second maxilla in the Zoea IV (Fig. 15b). This type of musculature we found also associated with the first maxilla and the first and second maxillipeds (data not shown). The maxillipeds of the Zoea IV also contain fully developed intrinsic musculature (Fig. 17a, b). The pereiopod anlagen of the Zoea IV contain undifferentiated tissue but not any striated musculature (Fig. 17d, g, h). However, at the megalopa stage, the pereiopods have become completely functional as walking legs (Fig. 3) and include fully developed striated musculature (data not shown; compare [24]).

#### Pleonal musculature

Starting with the Zoea I, each of the six pleomeres includes dorsal extensor muscles (DEM), which anteriorly are fan-shaped and flattened, and posteriorly copped (Fig. 4, 5, 17c, e), and also ventral flexor muscles (VFM; Fig. 17e). Both types of musculature increase in size and complexity from one larval stage to the next (Fig. 4). The pleopod anlagen in the Zoea I and Zoea II contain undifferentiated tissue inside (data not shown) and cross striated muscles are visible from the Zoea III onwards (Fig. 4). The first and sixth pleomeres are not equipped with any pleopods. In the Zoea IV, the pleopod musculature consists of three distinct muscle strands that extend into the pleopods. These muscles have further increased in size in the Megalopa (Fig. 4).

### Heart and circulatory system

The heart (H) is present at hatching and is positioned dorsally, directly underneath the dorsal spine. Micro CT data show that the volume of the heart gradually increases during larval development (Figs. 3 and 15c-d). In cross-sections of the Zoea IV, the heart displays a triangular shape. A very thin sheet of muscle tissue, the myocardium, forms its wall (Figs. 15b, d, and 16a). The heart is embedded within the haemolymph-filled pericardial cavity (PCav) underneath the integument of the dorsal carapace. Haemolymph from the pericardial cavity enters the heart via ostia in the myocardium (O; Fig. 15d). Similar to the arrangement in adult Brachyura ([73]), in the Zoea IV, we could identify three main arterial vessels that exit the heart: the anterior aorta (AA; also called dorsal medial artery), the posterior aorta (PA; also called the superior pleonal artery), and the descending artery (DA). The anterior aorta projects anteriorly towards the cephalic region to supply the median brain and eyestalk ganglia (Figs. 11a, b, 12a, b, 13a, 14a, and 16a).

At the position where the eyestalks attach to the cephalothorax, the anterior aorta abruptly bends ventrally in a 90 degrees angle to course towards the median brain (Fig. 10a, g, h). Before reaching the median brain, the anterior aorta widens to form an ovoid chamber, the auxiliary heart or cor frontale (CF), a structure that has been described in many adult decapod crustaceans [74]. The ophthalmic artery (OA) leaves the cor frontale anteriorly, towards the eyestalks (Figs. 9f and 10a, b). There, it splits into two branches that invade the left and right eyestalks (Fig. 9e`). The cerebral artery (CA) descends ventrally from the cor frontale to invade the median brain (Figs. 10i-k). In agreement with the description on adult crayfish provided by Chaves da Silva et al. ([75]), the stomatogastric ganglion (STG) is located within the cor frontale (Fig. 10I). In addition, we could identify the cor frontale muscles (CFM) based on the anatomical description provided by these authors (Figs. 10i, j).

A conspicuous tissue composed of small, darkly stained cells is located posteriorly to the cor frontale, but anteriorly to the epithelium of the cardiac stomach. Again, based on the description provided for adult crayfish, ([75], [76]), we tentatively identify this cluster of cells as representing the anterior hematopoietic tissue (HPS; Figs. 10k and 11b).

The posterior aorta leaves the heart posteriorly to supply the pleon with haemolymph (Fig. 17b, c). The descending artery leaves the heart ventrally (data not shown; for details see [24]) to pass through the ventral nerve cord (Fig. 17d, f). It continues ventrally as sternal or subneural artery to supply the ventral nerve cord and the developing cephalothoracic limbs with haemolymph.

### Ion-transporting and respiratory epithelia, excretory and secretory systems

#### Gills

In accordance with the study by Hong ([77]) we could not find any gill anlagen in the Zoea I. The first tiny gill buds emerge in the Zoea II and continue to grow in the Zoea III (data not shown). In the Zoea IV, the gill anlagen can be recognized as undifferentiated tissue at the proximal regions of the developing pereiopods (Gi; Figs. 6d, e, 7a and 17g). The gill anlagen of the pereiopods one to three become lamellate and functional during the metamorphosis to the Megalopa, the other anlagen become functional after moult to the first juvenile [77].

#### Branchiostegites

In adult brachyurans, the branchiostegites, i.e. the lateral carapace folds that form the branchial chamber, function as additional organs involved in the transport of gases and ions [78]. In decapod larvae, it has been suggested that the epidermis of the branchiostegites acts as an ion-transporting epithelium [43], [44], [79], [45]. In our preparations, the epidermis was artificially detached from the cuticle in the region of the branchiostegites (Bst; Figs. 17a-c, g) and the entire cephalothorax (e.g. Figs. 13a and 14a) possibly due to osmotic effects during fixation. Although we found indications that the epidermis might have a more complex structure in the region of the branchiostegites, compared to the other regions of the cephalothorax, we did not analyse these tissues any further to avoid misinterpretation due to the possible artefacts.

#### Antennal glands

In the Zoea IV, antennal glands (AG) are located on both sides of the cephalothorax close to the insertion of the second antenna (Figs. 12a, b and 13a). They are considered to function as larval excretory organs (review [3]; and [80, 81]). Corresponding to this organ’s organization in adult decapods [73], we could identify a coelomosac which receives the haemolymph (Fig. 12c). The coelomosac is connected to a labyrinth from where the urine passes to the bladder (Fig. 13c). The bladder extends dorsally and turns into the nephridial canal. The nephridial canal opens to the outside through a nephropore (Fig. 13c`).

#### Tegumental glands

In the Zoea IV, many tegumental glands (TeG) of the rosette type [32] are present, usually just beneath the surface of the integument. They consist of cells with a lightly stained cytoplasm (Fig. 11e), which are radially arranged around a small pore which most likely is a duct connecting the gland to the surface. Some of the cells displayed darkly stained inclusions. Here, we documented tegumental glands at the base of the rostrum (Fig. 10 F), embedded within the labrum (Fig. 11b, e), and proximally within the mandibles (Fig. 15a).

#### Y-organ

Based on the anatomical description provided in Höcker [82], we were able to localize the endocrine Y-organs in the Zoea IV. This organ is an inconspicuous accumulation of cells located laterally underneath the carapace and embedded within the epidermis of the branchiostegites (Fig. 16d). In an anterior-posterior direction, this organ is located roughly between the anlagen of the third maxilliped and the first pereiopod.

### Sensory systems

#### Compound eyes

In the Zoea IV, the compound eyes are located at the distal tip of the eyestalks (Fig. 9a) and are composed of numerous single units, the ommatidia. Each ommatidium consists of light guiding structures, the corneal lens (Cl, Fig. 9c, d) and the crystalline cone (Cry), formed by four cone cells (Fig. 9c`). Unfortunately, in our semi-thin sections, the cornea was detached from the underlying tissue due to poor fixation (indicated by arrows in Fig. 9c). The retinular cells that contain the photopigments jointly form the rhabdom (Rh, Fig. 9c). The rhabdomeres of the neighbouring ommatidia are optically isolated from each other by screening pigment cells (Pi). Between the proximal and the distal region of the ommatidia there is a separating layer of distal pigment (DP). The axons of retinular cells target the visual neuropils in the eyestalks (Fig. 9d). Compound eyes are already present at hatching and their developmental elaboration in *C. maenas* was described [25].

#### Aesthetascs

In the Zoea IV, the first antenna is equipped with chemosensory sensilla, the aesthetascs (AES; Fig. 10c`). In longitudinal sections of the first antenna, we localized aesthetascs that project from the distal segment of the antennae (Fig. 10c, d), most likely corresponding to aesthetascs number one, two and three following the terminology laid out in ([83]). Deeper within the antennae, clusters of cell somata belonging to olfactory sensory neurons (OSN) could be observed (circles in Fig. 10c). Bundles of dendrites (DE) project from the somata into the aesthetascs sensilla. In one section, a bundle of axons (Ax) was traced that projected from the OSN cluster proximally towards the median brain (Fig. 10d).

#### Putative anlage of the statocyst

In the Zoea IV, an accumulation of tissue is present embedded within the proximal part of the first antenna (Fig. 10b). It appears as an ellipsoid, multicellular complex surrounding a narrow cavity (Fig. 12e). This narrow, slit-like cavity is flanked, on both sides, by a cell layer, that is two to four cells thick. We tentatively identified this structure as the anlage of the statocyst although we did not find any statolith at this stage (see discussion).

#### Dorsal organs

In the Zoea IV, surface views generated from micro-CT scans show the external pores of the dorsal organs in a position corresponding to that described by Meyer et al. ([19]). The pores are located dorsally in front of the dorsal spine on both sides of the midline (Fig. 8a - black arrowheads) and close to the attachment sites of the mandibular adductor muscles. Semi-thin sections show that these pores are formed by conical indentions of the dorsal cuticle close to the anterior aorta (Fig. 8b, b’). These indentions extend deeper below the body surface but with the methods used here we could not trace any cellular material associated with the cuticular indentions (Fig. 8b”).

### Central nervous system

#### The brain of the Zoea IV

The brain or syncerebrum is composed of the eyestalk neuropils and the median brain (nomenclature according to [24] with modifications according to [84–86]). As is typical for the arthropod nervous system in general, the neuronal somata surround a core of synaptic neuropil. The eyestalk comprises the visual neuropils lamina (La), medulla (Me) and lobula (Lo; Figs. 5 and 9b), and the associated neuronal somata. The lamina is a thin neuropil layer located proximally to the retina (Re) of the compound eye from which it receives bundles of the photoreceptor axons (Fig. 9d). The neurites that link the lamina and medulla form a characteristic chiasm (Fig. 9b` - black arrow head). The hemiellipsoid body/medulla terminalis neuropil complex (HN/TM) is located most proximally within the eyestalks, and according to embryological data, is part of the protocerebrum [87]. This latter neuropil complex receives a direct input from the deutocerebral chemosensory lobes (DCL) via the projection neuron tract (see below). The eyestalk neuropils are connected to the median brain *via* the protocerebal tract (PCT; Figs. 5 and 9f) which includes the projection neuron tract (PNT; see below). The protocerebrum (PC) is the most anterior part of the median brain and consists of a large cluster of cell somata that surrounds the protocerebral neuropils anteriorly and dorsally (Figs. 9f and 10a, b). The central body (CB) is an unpaired, transversally extending neuropil embedded within the protocerebrum (Fig. 11c). The anterior dorsal cells cluster (ADC), a conspicuous cluster of neuronal somata, is located on top of the protocerebrum (Figs. 10j, k and 11c). The unpaired stomatogastric ganglion (STG) is located within the cor frontale (see above) and is flanked by the anterior dorsal cell cluster. This ganglion is located directly anteriorly to the cardiac stomach and provides an innervation for the entire gastric system (Figs. 10j, k and 11a). The deutocerebrum (DC), the second neuromere of the syncerebrum, receives input from the first pair of antennae. It is situated posteriorly to the protocerebrum and is dominated by the paired deutocerebral chemosensory lobes (DCL; previously called “olfactory lobes”; Figs. 11c, d and 12d). These are the primary chemosensory processing areas, which receive input from the olfactory sensory neurons on the first antennae and are located laterally within the deutocerebrum. The deutocerebral chemosensory lobes are dense, spherical neuropils that are ventrally accompanied by a large cluster of cell somata of olfactory interneurons from which neurites project into the neuropil. A characteristic bundle of axons leaves the DCL, the projection neuron tract (PNT; Fig. 11d). It projects medially, where its fibres form a characteristic chiasm and continue towards the eyestalks to terminate in the HN/TM complex (data not shown). The tritocerebrum (TC), the 3rd neuromere of the syncerebrum, caudally adjoins the deutocerebrum. Its bilateral halves are medially connected by the small tritocerebral commissure (TC), and in the frontal section plains, the tritocerebral neuropil (TN) is visible close to this commissure (Fig. 12a). The paired circumoesophageal connectives (CEC; Figs. 5 and 13a) flank the oesophagus on both sides, and the commissural ganglia (CG) are laterally associated with the connectives at the level of the oesophagus.

#### Ventral nerve cord

The circumoesophageal connectives link the median brain to the ventral nerve cord (VNC) which conforms to the ladder-like ground plan with paired ganglia, which longitudinally are joined by connectives and transversely by commissures. The neuromeres of the ventral nerve cord display the typical architecture with a central core of synaptic neuropil surrounded by a cortex of cell somata (Figs. 17a, b, d). From anterior to posterior, the mandibular neuromere can be distinguished followed by the neuromeres associated with maxilla 1 and maxilla 2 (compare Fig. 1b). Posteriorly, three neuromeres, associated with the maxillipeds, follow, in addition to five neuromeres associated with the developing pereiopods (walking limbs). The neuromeres form the first mandible down to the third maxilliped collectively are referred to as the suboesophageal ganglia in adults (compare [84]). All postoesophageal neuromeres down to the eight thoracic neuromere are fused to form a synganglion (yellow in Figs. 4 and 5) the segmental composition of which is, nevertheless, obvious from the ganglionic neuropil cores and the nerves extending into the periphery. The pleonal ganglia are posteriorly connected to this synganglion as additional part of the ventral nerve cord (Figs. 4 and 17e). They are smaller than the thoracic ones and have long conspicuous connectives.

#### Ontogenetic changes

The general layout of the nervous system in the earlier zoea stages closely resembles that described here in detail for the Zoea IV (Figs. 4 and 5). The basic structure of the central nervous system is already laid out in the first larval stage in which the suboesophageal and thoracic neuromeres are already condensed to form a synganglion. Yet, there is a gradual increase in size from stage to stage. The first metamorphosis to the Megalopa is not accompanied by any dramatic structural changes at the macroscopic level according to the micro CT scans, but again an increase in size of the individual components takes place (Fig. 4 and 5; and compare [24]). One visible difference is that the chain of pleon ganglia appears shorter and more condensed in the Megalopa than in the Zoea IV (Fig. 4).

## Discussion

### Digestive system

In adult brachyuran crustaceans, four regions of the digestive system can be distinguished: oesophagus, foregut, midgut, and hindgut (for references see Table 2). The oesophagus, foregut and the hindgut derive from the ectoderm and thus are lined by a thin cuticle. In adult crabs, the foregut is located dorsally in the cephalothorax, surrounded by the digestive gland. The midgut and hindgut possess blind ending tubules, the caeca. The hindgut ends with the anus.

**Table 2:** Book chapters and reviews summarizing the internal anatomy of adult decapod crustaceans

Organ system	
General anatomy	McLaughlin 1983 [168], Bell and Lightner 1988 [169], Felgenhauer 1992a [105], Forest 1994 [170], Vogt 2002 [171], Davie et al. 2015 [73]
Digestive system	Dall and Moriarty 1983 [69], Icely and Nott 1992 [70], Factor 1995 [172], Brösing 2010 [173], Watling 2013 [174], Saborowski 2015 [175], McLay and Becker 2015 [176]
Vascular system, circulation, hematopoetic tissues, haemolymph	Martin and Hose 1992 [177], 1995 [178], Söderhäll & Söderhäll 2001 [179], Lin and Söderhäll 2011 [180], Wirkner and Richter 2013 [101], Wirkner et al. 2013 [181], Mac Gaw and Reiber 2015 [182], Terwilliger 2015 [183], Söderhäll 2016 [184],
Gas exchange, ion regulation, excretion	Mantel and Farmer 1983 [185], McMahon and Wilkens 1983 [186], Taylor and Taylor 1992 [106], McMahon 1995 [187], Péqueux 1995[107], Charmantier et al. 2009 [188], Wirkner and Richter 2013 [101], Lignot and Charmantier 2015 [78]
Endocrine organs, glands and secretion	Cooke and Sullivan 1982 [189], Fingerman 1992 [190], Webster 2015 [191, 192]
Integument, setae and chromatophores	Felgenhauer 1992 [193], Horst and Freemann 1993 [194], Dillaman et al. 2013 [195], Garm and Watling 2013 [196], McNamara and Milograna 2015 [197]
Connective tissue	Mellon 1992 [198]
Muscle and neuromuscular system	Govind and Atwood 1982 [199], Chapple 1982 [200], Mellon 1992 [198], Govind 1995 [201], Atwood 2014 [202], Medler and Mykles 2015 [203], Mykles and Medler 2015 [204]
Eyes	Strausfeld and Nässel 1981 [205], Shaw and Stowe 1982 [206], Stavenga and Hardie 1989 [207], Warrant and Nilsson 2006 [208], Glantz 2014 [209]
Chemosensory, mechanosensory, and other sensory organs	Ache 1982 [210], Bush and Laverack 1982 [211], Govind 1992 [212], Atema and Voigt 1995 [213], Breithaupt and Thiel 2011 [214], Hallberg and Skog 2011 [114], Boxshall and Jaume 2013 [215], Garm and Watling 2013 [196], Derby and Weissburg 2014 [216], Mellon 2012 [217], 2014 [218], Lenz and Hartline 2014 [219], Lohmann and Ernst 2014 [220]
Central nervous system	Sandeman 1982 [221], Govind 1992 [212], Sandeman et al. 1992 [222], Harzsch et al. 2012 [124], Strausfeld 2012 [223], Loesel et al. 2013 [224], Sullivan and Herberholz 2013 [125], Schmidt 2016 [127], Sandeman et al. 2014 [126], Harzsch and Krieger 2018 [86]
Reproductive system	Krol et al. 1992 [227], Talbot and Helluy 1995 [226], Lopez Greco 2013 [225]

#### Mandibles and Foregut

In *Carcinus maenas*, many aspects of the adult organization are already present at hatching. Zoea larvae masticate food items with their strong mandibles, and then take up the food particles by peristaltic movements mediated by the lateral dilatator muscles attached to the oesophagus [3]. The complex gastric mill of the cardiac stomach continues to grind the food particles. In the Zoea IV, the presence of the well-developed stomatogastric ganglion that provides an innervation of the foregut suggests that the stomatogastric mill is able of complex movements. In other brachyuran larvae, it was shown that the teeth and denticles of the gastric mill continue to differentiate into the megalopa and juvenile stages so that the gastric mill gradually takes over the role of masticating food items. In the spanner crab *Ranina ranina*, for example, the mandible’s function switches from cutting and masticating in zoeal stages to cutting and crushing in the following stages and the mandibular incisor and molar process of the Megalopa degenerate [88]. A similar ontogenetic change was shown for larvae of the stone crab *Menippe mercenaria* [23] and in the gastric mill of the clawed lobster *Homarus americanus* in which these modifications coincide with the drastic change of habitat and diet that these larvae face after metamorphosis [17].

The ingested food passes from the cardiac stomach across the cardio-pyloric valve to enter the pyloric stomach, where the filter press, a complex filtration system made of setae and ampulla, is located. A complex filter press is already present in the Zoea I of *C. maenas* (data not shown). Similar observations were made for the first zoeal stages of the decapod species *Maja brachydactyla* [89], *Uca vocator,* and *Panopeus occidentalis* [90], which also possess a well-developed pyloric filter. On the contrary, early life history stages of other brachyuran species were reported to possess a less well-developed pyloric filter system in early zoeal stages (*Ucides cordatus*, [91]; *Dyspanopeus sayi*, [92]), and a pyloric filter appears to be absent in the facultative lecitotrophic larval stages of *Sesarma curacaoense,* which use reserves stored in a yolk sac for nutrition during their pelagic phase [93]. These studies collectively indicate that a well-developed pyloric filter may be an important adaptation for processing and mixing soft food particles suggesting that the gastric anatomy closely mirrors the food spectrum of a crustacean larva. At the same time, it has to be noted that the studies cited above used different methods to analyse the larval digestive systems so that we may be well advised to repeat some of these analyses using histological sections of plastic embedded specimens to standardize the methods.

#### Digestive gland

As in adult brachyurans, in the zoeal stages the liquid food that has passed through the filter press enters the midgut *via* two lateral openings towards the paired lobes of the digestive gland (also called the hepatopancreas or midgut gland). In adults, this complex glandular system is composed of numerous blindly ending tubules and is one of the largest components of the digestive system. It is involved in food absorption and transport, secretion of digestive enzymes, and storage of lipid, glycogen and a number of minerals (for reviews see Table 2). In the zoeal stages of *C. maenas* analysed here, the system is much less complex than in adults and consists of lobes (rather than tubules) that surround a central lumen. The digestive gland is already present at hatching (confirmed by micro-CT analysis). In the Zoea I and II, the digestive gland is nearly spherical in shape and possesses small protruding lobes. During development, these lobes enlarge gradually giving the organ a more and more complex appearance. In adult decapods, the tubules of the digestive gland consist of four cell types: E- (embryonic), F- (fibrillary), R- (resorptive), and B- (blister like) cells [71]. E-cells are located at the ends of the tubules and most likely act as precursor cells for the remaining three cell types, although there is not any consensus yet about the developmental sequence of cell transformation (compare [94, 95, 71]). We identified the four cell types in the midgut gland of the *C. maenas* Zoea IV, indicating a high degree of differentiation of this system in response to the diverse planktotrophic diet. The most conspicuous cell type seen in our histological sections were the R-cells that we identified based on their characteristic large lipid inclusions. These inclusions were even visible in intact specimens as orange droplets underneath the dorsal carapace and in the micro-CT analyses of all zoea stages. In our experiments, *ad libitum Artemia* sp. nauplii were offered as food. Thus they were able to accumulate substantial lipid reserves within their R-cells. Cells with large amounts of lipid inclusions in the medial portions of the digestive gland were also found in larvae of *Paralithodes camtschaticus* (Lithodoidea; [96]). In addition, the size and quantity of lipid droplets that *P. camtschaticus* larvae accumulated depended on the quality and type of food, as well as on rearing conditions (i.e. water temperature and illumination). Furthermore, similar abundant lipid inclusions within R-cells were also reported in early developmental stages of the American lobster *Homarus americanus* (Homarida; [97, 98]). In larvae of the spider crab *Hyas araneus* (Brachyura), the lipid inclusions in R-cells of the digestive gland show most distinct changes in ultrastructure in response to feeding regime. Therefore, they are an important indicator of the larval nutritional state [72] so that we conclude that the *ad libitum* feeding regime during our experiments caused an excellent nutritional state of the larvae as witnessed by the massive lipid accumulation.

### Musculature

#### Muscles of the cephalothoracic appendages

All zoeal instars of *C. maenas* displayed a prominent mandibular adductor muscle associated with the mandibles similar to *Cancer anthonyi* larvae [21]. This distinct muscle is responsible for inward movement of the mandible and hence for macerating food items that will then be further grinded with the gastric mill that shreds the food into even smaller particles. The exopods of the first and second maxillipeds have a natatory function in the zoeal stages, are equipped with long setae and contain elaborate intrinsic musculature. These exopods are lost during the first metamorphosis [60] and the maxillipeds become part of the feeding apparatus of the megalopa stage.

#### Muscles of the pleon

The extensor and flexor muscles within the pleon were already described by Trask [21] in the Megalopa of *C. anthonyi* in which it serves to flex and extend the pleon. As previously described for *C. maenas* [99] and supported by the present study, these muscles are already present in the earlier zoeal stages showing that the ability to flex the pleon is important for these early life history stages. Live observations of the feeding and swimming behaviour in larval stages of porcellanid crabs showed that zoeae catch living prey with the endopods of the maxillipeds and hold it with the flexed pleon and telson [100]. Furthermore, zoeae flip their pleon as an escape reflex (personal observations; and [3]) much like the caridoid escape reaction as described e.g. for adult Astacida or Homarida (“tail flip”). Well-developed dorsal extensor muscles and ventral flexor muscles are essential both for feeding in the zoeal stages and for swimming in the megalopa stage. The swimming appendages of the pleon, the pleopods, and their intrinsic muscles gradually develop in the successive zoeal stages and the Megalopa swims by beating the pleopods [100], a function which in the zoeal stages is accomplished by the exopods of maxillipeds one and two.

### Heart and circulatory system

Adult decapod crustaceans possess an open circulatory system in which the heart pumps the haemolymph *via* arteries across tissue spaces between the organs, the haemolymph lacunar system. After the passage across the respiratory organs, sinuses channel the haemolymph back towards the pericardium (for references see Table 2). In adult Brachyura, the dorsally located heart is a muscular chamber (also called ventricle), which is suspended by elastic ligaments within a second chamber, the pericardial cavity. The haemolymph enters the heart through paired ostia, three pairs in the case of Brachyura [73]. The ventricle pumps the haemolymph into five main arteries that target the major organ complexes: an unpaired anterior aorta (median brain and eyestalk neuropils), an unpaired posterior aorta (pleon), an unpaired descending artery (ventral nerve cord and thoracic limbs), and paired anterolateral arteries (digestive system, antennal glands). These vessels branch into fine capillaries that supply the target tissues.

Some major components of the adult brachyuran circulatory system such as heart, anterior and posterior aorta, and descending artery were already identified in Megalopae of *C. anthonyi* [21] and *C. maenas* [24]. Our results confirm these findings for the Zoea IV of *C. maenas.* In addition, we identified components of the haemolymph lacunar system. Furthermore, we traced previously not identified features of the arterial system in the Zoea IV, namely the cor frontale, a myoarterial formation known from many adult decapod crustaceans and specifically from Brachyurans which acts as an auxiliary heart [74]. This structure represents an aortic dilation with its own intrinsic muscles and functions in stabilizing the haemolymph flow towards the median brain and eyestalks including the visual neuropils and the compound eyes (review [101]). As in adult decapods [75], in the Zoea IV, the stomatogastric ganglion is located within the cor frontale. The ophthalmic artery leaves the cor frontale anteriorly, then splits into two branches that invade the eyestalks. The cerebral artery ventrally descends from the cor frontale to innervate the median brain. These results suggest that a strong haemolymph supply to the nervous system as well as to the cephalic sensory organs is an important feature already present in larval stages.

In decapod larvae the heart assumes its function during late embryogenesis [14, 28, 102, 103]. Our data suggest a gradual growth and increase of complexity of the larval circulatory system that most likely extends into the juvenile stage similar to the penaeid shrimp *Metapenaeus ensis* [103]. For example, in adult Decapoda, the heart is supported by trabeculae inside [104, 105] but we did not find such structures within the heart of the Zoea IV. Nevertheless, we detected slight constrictions at the ventral wall of the larval heart that might be anlagen of trabeculae.

### Ion-transporting and respiratory epithelia, excretory systems

#### Gills

In adult decapods, the gills serve as principal organs for respiration and ion regulation (for references see Table 2). Corresponding to the ground pattern of Brachyura (review [73]), adult *C. maenas* possess nine pairs of gills (GI - IX): MXP2 – one podobranch (GI), one arthrobranch (GII), MXP 3 – one podobranch (GIII), two arthrobranches (GIV, GV), P1 – two arthrobranches (GVI, VII), P2 – one pleurobranch (GVIII), and P3 – one pleurobranch (GIX). In brachyuran crabs, the anterior gills mostly accomplish gas exchange, whereas the transport epithelia, responsible for osmoregulation are located in the posterior gills (reviews [106, 107]). There is an increasing interest in the ontogeny of the osmoregulatory system in decapod crustaceans and the ecological implications of larval osmotolerance for aspects such as dispersal, population maintenance and invasive potential of a species [43–45, 108, 79]. The zoeal stages of *C. maenas* do not possess functional gills, only gill anlagen that gradually develop and are visible from the Zoea II onwards [43], [77]. The gill anlagen of the pereiopods one to three become lamellate and functional during the first metamorphosis to the Megalopa, and the other anlagen become functional during the second metamorphic moult [43, 77]. In *C. maenas,* only the Zoea I has limited osmoregulatory capacities whereas all other zoeal instars are primarily osmoconformers, which in too low or too high salinities may suffer severe osmotic stress [43]. The Megalopae display a limited capacity to hyper regulate while juvenile and adults are strong osmoregulators and can occupy habitats with low and fluctuating osmolarity. During ontogeny, *C. maenas* undergoes an osmo-physiological metamorphosis along with the morphological metamorphosis [43]. From a developmental point of view, the seemingly abrupt maturation of the gills during the metamorphic moult, which must be accompanied by changes in the pattern of haemolymph circulation in the entire system, is an interesting point for future research. Furthermore, another question that needs to be addressed is to what extend antennal glands participate in osmoregulation in the zoeal stages.

#### Branchiostegites

In adult brachyurans, the branchiostegites function as additional organs to transport gases and ions [78]. In decapod larvae, the epidermis of the branchiostegites has also been shown to act as an ion-transporting epithelium (see above; and [43–45, 79–81]). The brachyuran crab *Eriocheir sinensis*, in European waters is an invasive species which as an adult can effectively invade freshwater habitats. Contrary to *C. maenas* zoeal stages, early stages of *E. sinensis* already possess a moderate capacity to hyper-osmoregulate using the inner epithelium of the branchiostegites [44]. The Megalopa of this species is capable of moderately hyper-/hypo-regulating using the filaments of the posterior gills, and the juvenile crab and adults are euryhaline and have strong hyper-/hypo-regulating capacities, also using the posterior gills. These results, for some brachyurans, support the idea of an osmo-physiological metamorphosis between the planktonic zoeal stages, the semi-benthic megalopa stage, and the juveniles, and also indicate that the larval capability to osmoregulate is an essential adaptive trait for population persistence and range expansion.

#### Antennal glands

In adult brachyurans, the antennal glands at the base of each second antenna are major excretory organs. Besides excretion, it is generally accepted that they are also involved in the regulation of haemolymph volume, its acid-base balance, and ionic composition (reviews [73, 78]). They consist of a coelomosac that is involved in ultra-filtrating the supplied haemolymph [105]. The ultra-filtrate (or urine) passes through the labyrinth, a compartment composed of spongy tissue for protein and glucose reabsorption. Through the nephridial canal, the urine is then transported into a bladder and discarded through the nephropore at the base of the second antenna. In the crayfish *Astacus leptodactylus* and the clawed lobster *H. gammarus*, the larval antennal glands already display all cytological features necessary for a functionality [80, 81, 108]. A similar report is available for the palaemonid shrimp *Macrobrachium amazonicum* [79]. This paired organ and its major substructures are also present at least in the Zoea IV of *C. maenas*, as well as in adults, and could be recognized by micro-CT analyses and in histological semi-thin sections. We conclude that the antennal gland is already functional at this stage, because also the nephropore is present in the Zoea IV of *C. maenas*. In brachyuran crustaceans, the ontogeny of the antennal glands and possible functional changes across the double metamorphosis is not yet understood.

### Sensory systems

Brachyuran larvae show behavioural reactions to an impressive range of environmental stimuli including light, odorants, gravity, hydrostatic pressure, currents, temperature, salinity, and food concentration (reviews [9, 10]). They are also equipped with an array of chemosensory sensilla on their mouthparts to probe the chemistry of pray items [34]. Zoeae utilize combinations of many environmental cues to control their position within in the water column and by distinct vertical migration use tidal currents for off- and onshore transport (reviews [7, 11, 12]). In *C. maenas* larvae, such circa tidal rhythms were shown to be also driven by endogenous systems [109–111], and the implications of this behaviour for dispersal were analysed in this species [109, 112, 113]. Furthermore, it is well known that brachyuran larvae can detect chemical and tactile cues from the habitat to identify suitable sites for metamorphosis (reviews [4, 7, 9, 113]).

#### Compound eye

The compound eyes are the most conspicuous sensory organs of *C. maenas* larvae. Larvae hatch with unstalked eyes that possess apposition optics and this general optical design does not change during subsequent development [25]. After the moult to the Zoea II, the compound eyes are positioned on movable stalks. During ontogeny, the number of ommatidia and ommatidial length increases, which coincides with a decreasing interommatidial angle. These findings suggest a gradual increase of visual performance in terms of resolution and sensitivity during larval life and juveniles but not any fundamental change in eye design [25].

#### Aesthetascs

Aesthetascs are considered the most important unimodal chemosensory sensilla for distance chemoreception on the first antennae of adult decapod crustaceans (review [86]). In addition, malacostracan crustaceans possess a multitude of bimodal chemo- and mechanosensory sensilla associated with their two pairs of antennae, mouthparts and walking legs. A cluster of olfactory sensory neurons (OSNs) is associated with each aesthetasc. These bipolar neurons extend highly branched outer dendritic segments into the sensillum’s shaft and their axons project into the primary olfactory processing centres of the median brain, the deutocerebral chemosensory lobes (also called olfactory lobes). In *C. maenas,* Zoea IV larvae possess seven of these aesthetascs per antenna [83] and adults between 150 and 280 [86, 114]. Our current understanding is that the cluster of OSNs associated with one aesthetasc represents the animal’s full spectrum of functional olfactory receptor proteins and hence determines the olfactory landscape the organism is able to detect. This view suggests that even a Zoea can detect a similar range of odorants as an adult animal and that the increasing number of aesthetascs during ontogeny primarily increases the sensitivity of the olfactory system [86].

#### Dorsal organ

The sensory dorsal organ of crustacean larvae is at the dorsal side of the cephalothorax medially between the dorsal spine and the compound eyes. Externally it is marked by several pores, and internally consists of a both sensory cells and gland tissues [115–117]. In decapod larvae, this organ may act as a chemo- or mechano-/baro-receptor and its central pore may be associated with the gland tissue gland but such suggested roles need to be confirmed by functional studies [117]. Using scanning electron microscopy, the dorsal organ in the Zoea I stage of *Portunus acuminatus* was traced [19]. We were able to recognize the external pores of the dorsal organ. However, our histological methods were not suitable to identify any tissue associated with this organ. Thus, additional methods, such as transmission electron microscopy, are indispensable for further investigations of the cellular composition of the putative dorsal organ of *C. maenas* larvae.

#### Statocyst

Brachyuran crustaceans use statocyst organs within the second antennomer of the first antennae to detect gravity, angular acceleration, and hydrostatic pressure [118–121]. The statocyst of the prawn *Palaemon serratus* is sensitive to vibration and therefore used for sound reception [122]. The statocyst organ consists of sand granules embedded in a gelatinous substance, which is located on top of an array of sensilla at the floor of the statocyst. The sand granules function as a statolith and are renewed after each moult [123]. Little is known about the presence and development of a statocyst in crustacean larvae. The statocyst of the puerulus of *Jasus edwardsii* does not yet display any anatomical features such as the sensilla, secretory pores, and fluid within the statocyst cavity which are typical of the adult [120]. There is ample evidence from behavioural studies for geotaxis in brachyuran larvae (review in [10]) but the organ to detect gravity has not been identified. Our present study suggests a flattened ellipsoid cell accumulation at the base of the first antenna to be the anlage of a statocyst in the Zoea IV of *C. maenas*. The cell layers surround a small cavity, but a sensory epithelium with sensilla and a statolith could not be found suggesting that the statocyst anlage is not functional yet. Zoeal stages are planktonic and therefore do have little contact with sediment or inorganic granules to take up as statolith as may have been the case for the larvae used in our studies because they were cultured without any sediment. It remains to be explored which organ is used to detect gravity in pelagic larvae.

### Central nervous system and neuroendocrine system

#### Central nervous system

The general structure of the crustacean central nervous system (CNS) (reviews [124, 125]) and specifically the architecture of the brain (reviews [86, 126, 127]) is well understood in reptantian crustaceans including *C. maenas* [128]. Many CNS structures described in adult brachyurans are present in the late zoeal and megalopal stages [24]. Our micro-CT analysis now extend these descriptions for the Zoea I. Together with data presented for the Zoea I of *Pachygrapsus marmoratus* [30], our data on *C. maenas* suggest that already at hatching, the brachyuran CNS displays a remarkable degree of complexity and thus is able to perform sophisticated processing tasks. Nevertheless, the neuropils of thoracic neuromeres that are associated with non-functional limb anlagen in the zoeal stages, show strong postembryonic growth (e.g. [36, 37]). In addition, ganglionic elements tend to fuse and condense in larval and juvenile stages (review [84]) in the ontogenetic process of carcinization [73]. In addition to neuropil growth that characterizes the median brain [37], new neurons are also added to the ventral nerve cord during larval development [36, 38], as well as the visual neuropils [36, 41], and the olfactory pathway as determined by the use of mitosis markers [37].

#### Neuroendocrine system

Organs that are known to be of major importance for endocrine and neuroendocrine regulation during embryonic and larval development in reptantian crustaceans include the Y-organ, the mandibular organ, and the X-organ/sinus gland complex within the eyestalks (reviews [3, 129]). Our histological methods are not well suited to identify the developing neuroendocrine organs of the eyestalk. Nevertheless, by immunohistochemical localization of neuropeptides (moult-inhibiting hormone), some structural aspects of the X-organ/sinus gland complex in *C. maenas* larvae were described [130, 131]. Furthermore, following a previous anatomical description [82], we were able to identify the Y-organ. In adult decapods, this organ is described as a paired, spindle shaped gland anterior to the junction of the branchial and pre-branchial chamber, closely associated with the epidermis and surrounded by hemocoel [132], a description that closely fits to the structure which we identified as the Y-organ in the Zoea IV. The Y-organ was suggested to promote the proecdysis by release of ecdysteroid hormones [129], and is modulated by the release of moult-inhibiting hormones of the X-organ-sinus gland complex, the major neuroendocrine centre [3]. Collectively, these findings indicate that anlagen of all major neuroendocrine and hormonal organs of reptantian crustaceans are most likely present already at hatching [3, 129, 130].

### Advantages of multimethodological approaches

In our study, the combination of different methods facilitated the coherent analysis of the larval anatomy. The detection of auto-fluorescence has proven to be a fast and cost-efficient method assessing external morphology (e.g., [35, 133]) and allows to document specimens under high resolution without introducing artefacts by additional preparation steps. Furthermore, in contrast to techniques such as scanning electron microscopy the specimens can be used for additional histological experiments after imaging, and even anatomical details below the cuticle can be detected [133].

We showed that using micro-CT provided enough resolution for recognizing the most important organ systems in *C. maenas* larvae and to visualize their gross anatomy. Most importantly, micro-CT allows for reconstructing the spatial relationships of organ systems [134]. Our data demonstrate that this technique facilitates the anatomical analysis even of small arthropods and therefore provides a promising extension to the methodological spectrum used in previous studies on brachyuran larvae. The generation of anatomical atlases in invertebrates is traditionally based on serial sectioning of dissected tissues or entire organisms. This is a time-consuming procedure prone to artefacts such as section loss, distortion, and staining artefacts. Therefore, we suggest that non-invasive approaches such as micro-CT are likely to more accurately reflect the spatial arrangement of certain organs compared with invasive techniques and should therefore be favoured [134] although micro-CT analyses are prone to a certain amount of tissue shrinkage during the drying process [135]. Furthermore, obtaining micro-CT data is fast so that in the future we may be able to analyse organogenesis including volumetric data in larger numbers of specimens that were exposed e.g. to different experimental treatments during rearing.

Different tissues might possess equal X-ray densities and thus equal grey values in tomographies, which might hinder discrimination. In such cases, more details can be obtained by analysing semi-thin histological sections with a high resolution. This approach is time consuming but can complement micro-CT analyses in order to gain anatomical information down to the single-cell level [25]. For example, in our study, histology provided solid information on different cell types of the digestive system, which was not possible with micro-CT. Finally, immunohistochemistry provides yet another tool to analyse details of specific cell types such as ionocytes in the developing gills [43] or specific neuronal populations within the central nervous system [41, 42].

## Conclusions

The combination of imaging techniques used in the present study provided additional insights into the bewildering diversity of organ systems that larvae possess, which are necessary for autonomously surviving and developing in the plankton. This rich organ repertoire is crucial for generating adaptive behaviours and responding to variations in environmental key factors such as light, hydrostatic pressure, tidal currents, temperature, salinity, and food concentration (reviews [9, 7, 10]). Arguably, the histological complexity and behavioural repertoire of zoeae may be viewed as exceeding that of other arthropod larvae, e.g. those of holometabolous insects such as the vinegar fly *Drosophila melanogaster* and that of other crustaceans such as copepods which are in the same size range as adults.

Much of our fascination for the anatomy of brachyuran larvae stems from the opportunity to observe a complete and complex organism on a single microscopic slide and the realization that the entire decapod crustacean bauplan does unfold from organ anlagen compressed into a miniature organism in the sub-millimetre range. Despite their different life styles and outer morphology, brachyuran larvae are smaller versions of the adults when considering their inner organization. Along these lines, Trask [21] concluded that: “The major difference between the first, intermediate and last larval forms is basically one of size of the various systems involved, rather than absolute complexity”.

Despite the numerous examples for a seemingly gradual development of organ systems across the double metamorphosis that we discussed here, several of the metamorphic processes may nevertheless be promising targets for future investigations by developmental biologists:

- How is the musculature of the pereiopods refined during the seemingly abrupt transition to functionality during the first metamorphosis?
- Which processes in the central nervous system coincide with the maturation of the neuromuscular system in the pereiopods?
- How do the gills associated with the pereiopods become functional and supplied with haemolymph by the circulatory system during the first metamorphosis?
- Do the antennal glands change their function as the gills start functioning as osmoregulatory structures after the first metamorphosis?

Contrary to holometabolous insects for example, these metamorphic transformations must occur “on the fly” in an organism that autonomously must find food, that is constantly exposed to predation and must respond to tidal currents. For many decades, brachyuran larvae have served as distinguished models in the field of Ecological Developmental Biology (reviews [4, 7]). Our study may provide a means to link such ecophysiological studies to the level of tissues and organs.

## Methods

### Handling of berried females and larval rearing

Berried females of the European shore crab *Carcinus maenas* Linnaeus, 1758 (Decapoda, Brachyura, Portunidae), were collected at the western intertidal of the island of Helgoland (Germany) during their reproductive period. To avoid possible acclimation effects to laboratory conditions, only females with eggs in late embryonic stages (dark grey-brown coloured) were chosen thus ensuring that most of the embryonic development occurred within the natural habitat. Females were transported to the Biologische Anstalt Helgoland (BAH) of the Alfred Wegener Institute, Helmholtz Centre for Polar and Marine Research (Germany). They were kept at 18 °C in individual, aerated 20 L aquaria, filled with natural seawater, corresponding to the conditions in their native habitat in summer. Females were fed twice a week with frozen shrimps (*Crangon crangon*) and the water was changed daily in order to ensure high water quality at hatching. After hatching, freshly hatched larvae (Zoea I) were transferred to 400 ml glass-bowls (50 animals per bowl) filled with filtered natural seawater and kept at 18 °C [43]. Larval numbers were decreased after each successive stage, Zoea II, III, IV and Megalopa, to 40, 30, 20, and ten individuals per bowl, respectively, to account for growth. Water and food (freshly hatched *Artemia* sp. nauplii *ad libitum*) were exchanged daily. During each water change, dead larvae were discarded and moulted larvae were transferred to a separate bowl to ensure that the sampled larvae were at the same instar. Each larval stage was sampled at intermoult (i.e. when 50% of each moult cycle had occurred) according to preliminary data on larval developmental times (F. Spitzner, unpublished data).

### Imaging of cuticular autofluorescence

For autofluorescence imaging, Zoea IV were fixed in 70% ethanol and stored within the fixative until further use. For microscopic analysis, single larvae were transferred onto microscopic slides, submersed in fresh ethanol and cover slipped using modelling clay as spacers. Whole mounts were viewed using a Nikon Eclipse 90i upright microscope equipped with a digital camera by using appropriate filter sets (excitation 359-371 nm; emission 379 nm).

### Preparation for X-ray micro-computed tomography

For reconstructions of the internal anatomy using X-ray micro-computed tomography (micro-CT), specimens of each zoeal stage were fixed in Bouin‘s solution (saturated aqueous picric acid, concentrated acetic acid and 10 % formaldehyde solution) and stored in the fixative until further analysis. To ensure adequate fixation, the dorsal and rostral spines, as well as the pleon were dissected. For micro-CT analysis, samples were processed according to [134]. Fixed specimens were washed three times in sodium hydrogen phosphate buffer (0.1 M Roti®fair PBS pH 7.2 [Carl Roth] with 1.8 % sucrose) and dehydrated *via* a graded ethanol series. Afterwards, samples were incubated overnight in a 1 % iodine solution (iodine, resublimated [Carl Roth #X864.1] in 99.8% ethanol) and washed three times in ethanol (99.8%). Specimens were critical point dried with an automated dryer Leica EM CPD300 (Leica Microsystems GmbH, Wetzlar, Germany) and subsequently fixed on insect needles with hot glue. The samples were scanned with an Xradia MicroXCT-200 X-ray imaging system (Carl Zeiss Microscopy GmbH). Tomographic scans were obtained using a 10x magnification lens unit with X-ray source settings at 40 kV and 200 μA, and with 1.5 s acquisition time. The reconstruction of tomography projections was processed by using the XMReconstructor software, resulting in tiff-format image stacks.

### Three-dimensional reconstruction

Segmentation and volume rendering of image stacks was performed by using the software Amira 5.4.5 and Amira 5.6.0 (FEI Visualization Science Group, Burlington, USA). Overall, three replicates of each zoeal stage were analysed in order to render the volume of the nervous system, digestive tract, heart and musculature. Using every second slide, the respective organ system of each zoeal stage was reconstructed as described by in [134]. Afterwards, volume renderings were performed by using the “Volren” function. For images of the dorsal organ, a volume rendering, generated with the “Volren” function of Amira, of the outer surface of a Zoea IV was used.

### Semi-thin sectioning

For histological sections, larvae of the fourth stage were immersed in FAE (80% ethanol, 37% formalin, pure acetic acid), following dissection of limbs and pleon, and stored in the fixative until further analysis. After washing in several changes of PBS (0.1M, pH7.2, 1.8% sucrose), specimens were post fixed for 1 hour in osmium tetroxide. After washing in three changes (20 min. each) of PBS, specimens were transferred to 30% acetone and dehydrated through an ascending series of acetone to 100%. The dehydrated samples were transferred to a 1:1 mixture of acetone: araldite (Araldite epoxy resin kit, Agar Scientific) overnight. The larvae were then embedded in pure araldite and incubated for 2 days at 60 °C for polymerization. Embedded larvae were sectioned (1.5 μm) with a Hyrax S50 vibratome (Zeiss). Finally, sections were stained after Holländer and Vaaland [136] with a solution of 1% phenylendiamin in methanol-isopropanol for 12h. Afterwards, the slides were covered with Roti®-Histokitt and cover slips. Sections were viewed using Nikon Eclipse 50 and 90i upright microscopes equipped with a digital camera (Nikon Digital Sight DS 2MBWc). The section plane of the selected sections in Figs. 9, 10, 11, 12, 13, 14, 15, 16 and 17 is shown in Fig. 2c.

### Nomenclature

If not otherwise indicated, we will use the general anatomical nomenclature as proposed in publications [3] and [73]. The central nervous system has already been described in some detail for the Megalopa of *C. maenas* [24]. Because the general layout of the nervous system in the zoea stages resembles that of the Megalopa (Figs. 5 and 6), we will use the nomenclature proposed in [24] with significant modifications according to [84–86].

## Abbreviations

A1: first antenna
A2: second antenna
AA: anterior aorta
AC: anterior caeca
ADC: anterior dorsal cells of the protocerebrum
AES: aesthetasc
AG: antennal gland
AGM: anterior gastric muscles
AL: anterior lobe
AR: ampullary ridge
ASS: ampullary setal screen of the gland filter
Ax: axon
B: blister like cell
Bl: bladder
Br: median brain
BSt: epithelium of branchiostegite
CA: cerebral artery
Cap: capillary
CB: central body
CEC: circumoesophageal connective
CF: cor frontale
CFM: cor frontale muscle
CG: commissural ganglion
CL: corneal lens
CMsc: contractor muscles of cardiac stomach
Co: cornea of compound eye
CoeSac: coelomosac
Cry: crystalline cone
CS: cardiac stomach
CSac: cardiac sac
Cu: cuticle
CV: cardio-pyloric valve
DA: descending artery
Da: dactylus (in Fig. 2)
DC: Deutocerebrum
DCL: deutocerebral chemosensory lobes
De: dendrites
DEM: dorsal extensor muscles
DG: digestive gland
DGM: dorsal gastric muscles
DL: dorsal lobe
DLC: dorsolateral pyloric channel
DMsc: dilatator muscle of esophagus
DP: distal pigment
DPC: dorsal region of the pyloric chamber
DPF: dorsal pyloric fold
DS: dorsal spine
E: embryonic cell
Eso: oesophagus
Ey: compound eye
Ey stalk: eyestalk
EyNP: eyestalk neuropil
F: fibrillary cell
FG: foregut
FP: filter press
Gi: gill
H: heart
HG: hindgut
HN/TM: complex of hemiellipsoid body neuropil and medulla terminalis
HPT: hematopoietic tissue
IAR: interampullary ridge
IP: incisor process
La: lamina
LAC: lower ampullary chamber
Lb: labrum
Lo: lobula
LT: lateral tooth
Lu: lumen of the digestive gland
Ly: Labyrinth
MA: mandibular adductor muscles
Md: mandible
MdP: mandibular palp
Me: medulla
MG: midgut
MP: molar process
Msc: musculature
MT: median tooth
Mx1: first maxilla
Mx2: second maxilla
Mxp1: first maxilliped
Mxp2: second maxilliped
Mxp3: third maxilliped
Neu: neurites
No: nephropore
NP: neuropil
O: Ostium
OA: ophthalmic artery
Om: ommatidium of compound eye
OSN: olfactory sensory neurons
P: pereiopods or pereiopod anlagen
PA: posterior aorta
PC: protocerebrum
PCav: pericardial cavity
PCT: protocerebral tract
PI: shielding pigment
Pl: pleopod anlagen
PLG: pleon ganglia
PLM: pleopod musculature
PM: pleomere
PNT: projection neuron tract
PoC: posterior caecum
PosL: posterior lobe
PPO: prepyloric ossicle
Pr: propodus
PS: pyloric stomach
PSS: pyloric setal screen
R: resorptive cell
Re: retina
Rh: rhabdom of photoreceptor cell
Ros: rostrum
RS: rostral spine
SA: sternal artery
SEG: suboesophageal ganglia
St: putative anlage of the statocyst
StG: stomatogastric ganglion
StN: stomatogastric nerve
T: thoracomere
TC: tritocerebrum
TeG: tegumental glands
TG 1-5: thoracic ganglia one to five
Tr: primary trabeculae
UAC: upper ampullary chamber
VFM: ventral flexor muscles
VGM: ventral gastric muscles
VL: ventral lobe
VNC: ventral nerve cord
Y: Y-organ
